# PIXUL-ChIP: integrated high-throughput sample preparation and analytical platform for epigenetic studies

**DOI:** 10.1093/nar/gkz222

**Published:** 2019-03-30

**Authors:** Karol Bomsztyk, Daniel Mar, Yuliang Wang, Oleg Denisenko, Carol Ware, Christian D Frazar, Adam Blattler, Adam D Maxwell, Brian E MacConaghy, Thomas J Matula

**Affiliations:** 1 UW Medicine South Lake Union, University of Washington, Seattle, WA 98109, USA; 2 Institute for Stem Cell and Regenerative Medicine, University of Washington, Seattle, WA 98109, USA; 3 Paul G. Allen School of Computer Science & Engineering, University of Washington, Seattle, WA 98195, USA; 4 Department of Genome Sciences, University of Washington, Seattle, WA 98195, USA; 5 Active Motif, Carlsbad, CA 92008, USA; 6 Department of Urology, University of Washington School of Medicine, Seattle, WA 98195, USA; 7 Center for Industrial and Medical Ultrasound, Applied Physics Laboratory, University of Washington, Seattle, WA 98195, USA

## Abstract

Chromatin immunoprecipitation (ChIP) is the most widely used approach for identification of genome-associated proteins and their modifications. We have previously introduced a microplate-based ChIP platform, Matrix ChIP, where the entire ChIP procedure is done on the same plate without sample transfers. Compared to conventional ChIP protocols, the Matrix ChIP assay is faster and has increased throughput. However, even with microplate ChIP assays, sample preparation and chromatin fragmentation (which is required to map genomic locations) remains a major bottleneck. We have developed a novel technology (termed ‘PIXUL’) utilizing an array of ultrasound transducers for simultaneous shearing of samples in standard 96-well microplates. We integrated PIXUL with Matrix ChIP (‘PIXUL-ChIP’), that allows for fast, reproducible, low-cost and high-throughput sample preparation and ChIP analysis of 96 samples (cell culture or tissues) in one day. Further, we demonstrated that chromatin prepared using PIXUL can be used in an existing ChIP-seq workflow. Thus, the high-throughput capacity of PIXUL-ChIP provides the means to carry out ChIP-qPCR or ChIP-seq experiments involving dozens of samples. Given the complexity of epigenetic processes, the use of PIXUL-ChIP will advance our understanding of these processes in health and disease, as well as facilitate screening of epigenetic drugs.

## INTRODUCTION

The chromatin immunoprecipitation (ChIP) assay, a widely-used approach for identifying histone modifications and genome-associated proteins, is one of the most powerful tools to study transcription and epigenetics processes (1–6). We have previously developed a high-throughput microplate ChIP assay, Matrix ChIP, which speeds up the analytical process, dramatically increases the assay's throughput, and provides superior sensitivity and reproducibility as compared to other protocols (7–9). Although the introduction of Matrix ChIP and other high-throughput ChIP platforms (10,11) was a major improvement, their utility was limited by low throughput and efficiency of the existing methods for chromatin sample preparation.

The most common approach used for chromatin fragmentation is ultrasound treatment. Ultrasound waves transmitted into liquids generate cycles of alternating high pressure (compression) and low pressure (rarefaction), with rates governed by the applied frequency. The rarefaction phase creates cavitation, in which vapor and/or gas bubbles expand and then collapse violently. Cavitation in liquids has many applications including chromatin sample preparation for ChIP (12). Enzymatic digestion is alternatively used for chromatin fragmentation (6) but conditions vary depending on the application (13). Enzymatic digestion may also require ultrasonic pre-treatment (14,15) especially for tissues. Recently, targeted *in situ* enzyme-based genome-wide profiling methods have been introduced but these use un-fixed chromatin (16,17). There are a number of different commercially available ultrasound instruments that use cavitation to shear chromatin, including microtip probes, horns, water bath- including microplate-based methods. As none of these sonication instruments can be directly applied to culture plates, harvesting of cells and their transfer to tubes or plates is inefficient, resulting in sample losses. Further, the commonly used Covaris sonicators use expensive tubes or 96-well plates that cost more than $400/plate. To match the high-throughput capacity of microplate ChIP analytical platforms (8,9,11,18), we developed an instrument, PIXUL, that consists of an array of ultrasound transducers that shear chromatin in each and all wells using off-the-shelf low cost 96-well plate (∼$2/plate). We integrated PIXUL with ChIP, PIXUL-ChIP, for high-throughput transcription and epigenetic analysis of cultured cells and tissues. We also provide examples that PIXUL has the potential to be used as a multipurpose sample preparation platform.

## METHODS

### PIXUL instrument components (Supplementary Figure S1)

#### Ultrasound treatment system

PIXUL is custom-built and comprises the following main parts: (i) a transducer-lens assembly capable of focusing ultrasound in each well of a 96 well microplate, (ii) a high power amplifier to drive the transducer array, (iii) a Peltier cooling system to reduce heating of the samples and (iv) a computer to control the ultrasound pulse parameters (number of cycles, treatment configurations and treatment time) (Supplementary Figure S1).

The transducer array is composed of flat lead-zirconate-titanate (PZT) ceramic bar segments bonded to the base of a 96-element lens array such that each lens focuses acoustic energy into an individual well of the microplate (patent pending, WO 20170205318). The operating frequency is approximately 2 MHz. The transducers are driven with a high voltage pulse from the amplifier. The bonded lens focuses the ultrasound, creating intense cavitation in the sample fluid as well as vigorous mixing during sonication. In free field, each focused transducer element produces up to 30 MPa peak positive (–12 MPa peak negative) pressure.

The amplifier is a purpose built multi-channel high power amplifier (19) capable of applying sufficient voltage to the transducer array to generate the required intense cavitation in the sample wells. The amplifier system consists of an FPGA (field-programmable gate array) timing board, high-voltage switching boards, and an external power supply. The timing board controls the ultrasound pulsing parameters, which are programmed through USB using MATLAB software (MathWorks, Natick, MA, USA) on a standard computer. Matching networks are included between the output of the amplifier and transducer to maximize power transfer. The timing board operates the high voltage switching boards to create a high-voltage signal that is applied to the transducers.

The Peltier-cooled liquid (water and glycerol mixture) flows (at ∼40 ml/min) between the transducer/lens array and the bottom of the microplate and acts to couple the ultrasound to the microplate samples, and also to reduce sample heating.

### Cell lines and treatment

Cells were grown in round-bottom 96-well polystyrene plates. Human HCT116 colon carcinoma and human HEK293 kidney cell lines were grown (∼200 000/well) in DMEM supplemented with glutamine, penicillin, streptomycin and 10% fetal bovine serum. For time-point experiments, cells were serum-deprived (0.1% FBS) overnight and at specific time points were treated with either 10% FBS or 12-tetradecanoate 13-acetate (TPA) at 10^−7^ M.

### Cell cross-linking, harvesting and sonication

All PIXUL steps were done directly in 96-well culture plates without sample transfers. Cells were cross-linked by adding 100 μl 1% formaldehyde in PBS to overlaying media, and the plate was shaken for 15 s. Cross-linking was done for 20 min at room temp. Supernatant was removed, 200 μl PBS/glycine (125 mM) was added for 5 min at RT, and the wells were washed with 200 μl PBS (8,9,20). After removing PBS, wells were filled with 100 μl chromatin shearing buffer, and plates were sealed with PCR film (MiniAMP Optical Adhesive Film (Applied Biosystems, #4311971)) and placed in PIXUL for shearing. PIXUL was programmed to sonicate (50 cycles, 550 Pulse Repetition Frequency (PRF), 40 W) one pair of columns (8 + 8 wells) for 10 s and then electronically skip to the next pair (the plate is not mechanically moved). After all six pairs of columns were sheared, this sequence was repeated 24–36 times (total ON shearing time 4–6 min/well, 18–36 min/plate). Comparison of Covaris LE220 instrument with PIXUL was done using HCT116 cells cultured in 96-well plates as follows. After cross-linking (as above), cells from three wells were combined into one sample (one well was not sufficient for ChIP-qPCR) for shearing in either Covaris microplate or tubes, with total time 5 min/column (8 samples) (200 cycles, Duty Factor 15%, 450 W). In-well temperature was monitored with a small-size thermocouple (52II Thermometer, FLUKE). In-well start temperature with PIXUL was 4°C and slowly increased to 24°C at the end of the treatment (tank temperature remained at ∼14°C). In-well start temperature with Covaris was 7°C, increasing within one min to 20°C and reaching 22°C by the end of the 5 min treatment (tank temperature remained at ∼4°C).

Comparative studies using Bioruptor were done in 0.5 ml microfuge tubes as previously described (8,9). Bioruptor in-tube temperature could not be monitored, but tank starting temperature was 4°C and maintained <25°C during the run by stopping the treatment and letting the circulating water chill (circulating through ice bucket). Sonicated chromatin generated with each instrument was assessed by agarose gel electrophoresis and Matrix ChIP analyses.

### Mouse tissue cross-linking and sonication

Female and male 12-week-old WT (C57bl6) mice were used. Mice were euthanized by isoflurane overdose followed by cervical dislocation. Hearts, kidneys, livers and lungs were recovered, flash-frozen and stored at −80°C. All procedures were done in accordance with current NIH guidelines and approved by the Animal Care and Use Committee of the University of Washington.

Small tissue fragments (30–50 mg) cut from frozen organs were added to 1.5 ml centrifuge tubes containing 0.5 ml 1% formaldehyde in PBS and briefly homogenized with loose pestle motor mixer. After 20 min of cross-linking, formaldehyde was replaced with 0.5 ml 125 mM glycine/PBS for 5 min to quench the reaction, followed by PBS wash. Tissues samples from all these organs were then resuspended in 100 μl chromatin shearing buffer and added to wells of the 96-well plate for sonication in PIXUL using the same protocol as for cell culture.

### Matrix ChIP: multiplex microplate-based chromatin immunoprecipitation

The multiplex microplate Matrix ChIP method was previously described (8,9). Briefly, ChIP assays were done using protein A-coated 96-well polypropylene microplates (8). 1 μl of isolated DNA was used in 2 μl real-time qPCR reactions (done in 384-well plates using ABI7900HT). All PCR reactions were run in quadruplicate using Sybr green. PCR calibration curves were generated for each primer pair from a dilution series of total mouse or human genomic DNA. The PCR primer efficiency curve was fit to cycle threshold (Ct) versus log(genomic DNA concentration) using an *r*-squared best fit. DNA concentration values for each ChIP and input chromatin DNA sample was calculated from their respective average Ct values. Final results are expressed as fraction of input DNA (9). The list of ChIP antibodies is shown in Table S1 (supplement) and PCR primers in Supplementary Tables S2 and S3.

### RNA extraction and cDNA synthesis

RNA was isolated using TRIzol as per manufacturer's protocol. To synthesize cDNA, 400 ng of TRIzol-extracted total RNA was reverse transcribed with SuperScript IV (Invitrogen, 18090050), 0.2 mM dNTP (GeneScript, 95040-880) and oligo dT primers (IDT) in 10 μl reactions in 96-well microplates. RT reactions were diluted 100-fold prior to running qPCR. RT-qPCR primers are listed in Supplementary Tables S2 and S3.

For RNA extraction using PIXUL, small tissue fragments were added to wells of 96-well plates containing 100 μl TRIzol, and samples were treated using the same parameters as for chromatin sharing (50 cycles, 550 PRF, 40 W for 1 min/column of one organ). After PIXUL treatment, the rest of the procedure was same as for the standard TRIzol and RT-qPCR protocol.

### PIXUL-ChIP-seq

HCT116 cells were grown to a density of ∼200 000 cells per well, cross-linked, quenched and sonicated using 96-well PIXUL for 6 min per well in 100 μl. ChIP and library preparation were done using Low Cell ChIP-Seq Kit (Catalog number 53084, Active Motif, Carlsbad, CA, USA). Libraries were sequenced on a NextSeq 500 as PE75 with dual 8bp indexing, allowing for PCR de-duplication with molecular identifiers at the i5 position (as per product manual, https://www.activemotif.com/documents/2073.pdf).

ChIP-seq data was aligned to hg19 using BWA (version 0.7.12) (21). For each of the seven PIXUL-ChIP-seq data sets, corresponding ENCODE BAM files were downloaded from the ENCODE website (https://www.encodeproject.org) (22). If there were multiple experiments for the same epitope from different labs, we chose the one from the Bernstein Lab. Peaks were called using MACS2 (23) with the parameters –broad -broad-cutoff 0.1. Low quality peaks were filtered out as follows: for H3K4m1, H3K27m3 and H3K27Ac, peaks with q-value <1e–3 and fold enrichment >2 compared to input were kept. For all other histone marks, peaks with *q*-value <1e–10 and fold enrichment >5 were kept. Bedtools (24) (https://bedtools.readthedocs.io/) were used to identify peaks overlapping between PIXUL-ChIP-seq dataset and ENCODE.

Specialized source code generated for ChIP-seq analyses is available using the following link

https://github.com/yuliangwang/PIXUL_ChIP

PIXUL-ChIP-seq data can be viewed in genome browser using the following link

https://tinyurl.com/y9sap4qd

#### qPCR data

To acquire, store and analyze large qPCR data sets generated by the high-throughput Matrix ChIP platform, we used our previously developed graphical tool, PCRCrunch (7). Pair-wise statistically significant differences are represented on graphs by the size of a circle for each comparison, with a small circle representing *P* < 0.05, a large circle indicating *P* < 0.01 and no circle implying *P* > 0.05. PCRCrunch uses a two-tailed Student's *t*-test to compute *P*-values (7).

#### Agarose gel electrophoresis image processing software tool

Matlab 2017 with Signal Processing and Curve Fitting Toolboxes was used. The program utilizes a gel electrophoresis image to quantify both the relative concentration and the base-pair length of the DNA band in each well. First, the program converts original image into a gray color scale and resizes it to a linear scale (using the base-pair ladder from the gel to calibrate). Second, it goes through each well from the modified image and plots the normalized signal intensity as a best-fit curve, providing both the mean base-pair length and the percentage of signal that falls between 200 and 600 base-pairs in length (indicating the target shearing sizes). Finally, the program produces a waterfall plot that contains best-fit curves in sequential order (lanes 1–12) to compare the relative shapes and intensities of the DNA bands. The code has been deposited and is available at this link: https://github.com/kbomsztyk/Agarose-Gel-Electrophoresis-Image-Processing

DNA fragment size distribution measured by agarose gel electrophoresis method was compared to two commercial systems, Agilent Bioanalyzer (Agilent 2100) and Fragment Analyzer (formerly Advanced Analytical, now marketed by Agilent) (Supplementary Figure S2). The Bioanalyzer analyzes the biomolecules as they are electrophoresed through a microchannel in a glass chip that is primed with a gel/dye mix specific for the particular biomolecules being analyzed. The Fragment Analyzer separates biomolecules based on capillary electrophoresis. The mean size measured with the agarose gel system was ∼25 bp smaller than that measured using Agilent Bioanalyzer but 40 bp larger than that measured with Fragment Analyzer. (The difference between the two Agilent instruments was ∼70 bp) (Supplementary Figure S2F). The agarose gel fragment distribution between gDNA replicates was close to those measured with Fragment Analyzer and Agilent Bioanalyzer (Supplementary Figure S2). These comparisons show that the agarose gel electrophoresis system is well suited to analyze size distribution of sheared DNA fragments, provided that they can be visualized on gel with a camera.

## MATERIALS

Proteinase K (25530-015) was from Invitrogen. Bovine serum albumin (BSA, A9647), salmon sperm DNA (D1626), transfer RNA (tRNA, MRE600), and protein A (P7837) leupeptin (L2884), β-glycerophosphate (G6251), sodium fluoride (NaF,S1504), sodium orthovanidate (Na3VO4, S6508), phenylmethyl sulfonylfluoride (PMSF P7626), dithiothreitol (DTT, D0632), p-nitrophenyl phosphate di(tris) salt (N3254), sodium molybdate dihydrate (Na_2_MoO4 • 2H20, S-6646), EDTA (E3134), Tris–HCl (T3253) were from Sigma. Sodium chloride (NaCl S-271-3) and Triton X-100 (BP151) was from Fisher. Formaldehyde (28908) was from ThermoFisher. NP40 (198596) from MP Biomedicals. McCoy's medium (SH3020001) and Dulbecco's Modified Eagle Medium (DMEM- SH30021.0) were from HyClone, penicillin/streptomycin (P/S 15749) from Invitrogen, fetal bovine serum (FBS 43635-500) from Jr. Scientific, and phosphate buffered saline (PBS 70013-032), TRIzol (15596018) from Life Technologies. Labware and kits catalog numbers, commercial suppliers and costs are listed in Supplementary Table S4.

## RESULTS AND DISCUSSION

### Chromatin and DNA ultrasound treatment in PIXUL

**Figure 1.**
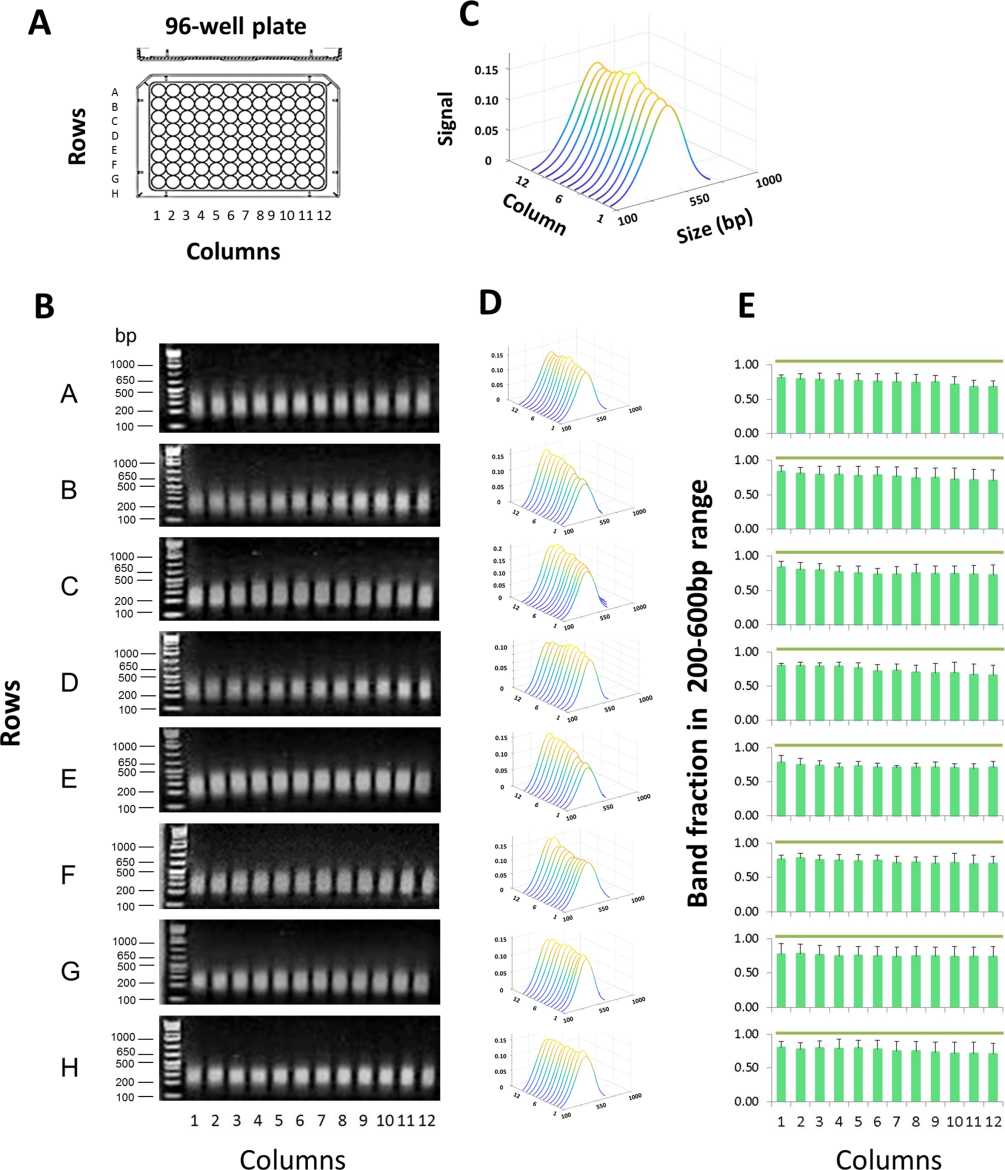
PIXUL shearing of DNA in 96-well plates. (**A**) Shearing was performed in 96-well plates (with each well containing salmon DNA at 100 ng/μl in 100μl volume/well) for a total treatment time of 36 min per each plate. (B) Agarose gel electrophoresis of DNA fragments, gels were stained with ethidium bromide. DNA ladder was run in the first lane of each gel. Numbers to the left of the gels show sizes of selected ladder bands in base pair (bp). (**C**) An example to illustrate a waterfall plot (MATLAB) with annotated axis. Image software was used to analyze stained DNA bands (Methods). Results represent best-fit curves in sequential order of samples from PIXUL plate column wells 1 to 12. X- axis; band size in base pair (Size (bp)). Y-axis; sample from a well of a given column (columns 1–12). Z-axis; relative signal intensity of DNA bands for given plate well (Signal). (**D**) Waterfall plots for each plate row (rows A through H). (**E**) Graphs represent band fraction in the 200–600 bp range from each one of the 96 wells (mean ± SDEV, *n* = 3 experiments). These results demonstrate consistent DNA shearing across all wells of a 96-well plate.

PIXUL development was aimed to make an array of ultrasound transducers with identical performance across all 96 wells that utilize low-cost, off-the-shelf consumables. To estimate ultrasound treatment efficiency without the confounding effect of DNA crosslinking, we first used purified salmon DNA, which is readily available in large quantities. 100 μl of salmon DNA at 100 ng/μl was aliquoted into each one of the 96 wells of two replicate plates. After sealing wells with tape, the plates were treated with ultrasound in PIXUL (total time 36 min per plate). Sheared DNA fragments were assessed by agarose gel electrophoresis and ethidium bromide staining. Gel images were analyzed using the in-house-developed MATLAB-based agarose gel electrophoresis image analysis software tool as described above (Methods). Figure 1 demonstrates similar size distribution of DNA in all 96 wells. Across all wells, the average size of the sheared fragments was 307 ± 35 bp and on the average, 74.6 ± 3.7% of the band fragments were within the 200–600 bp size range (mean±SDEV, *n* = 3, 96-well plates) (Figure 1E).

**Figure 2.**
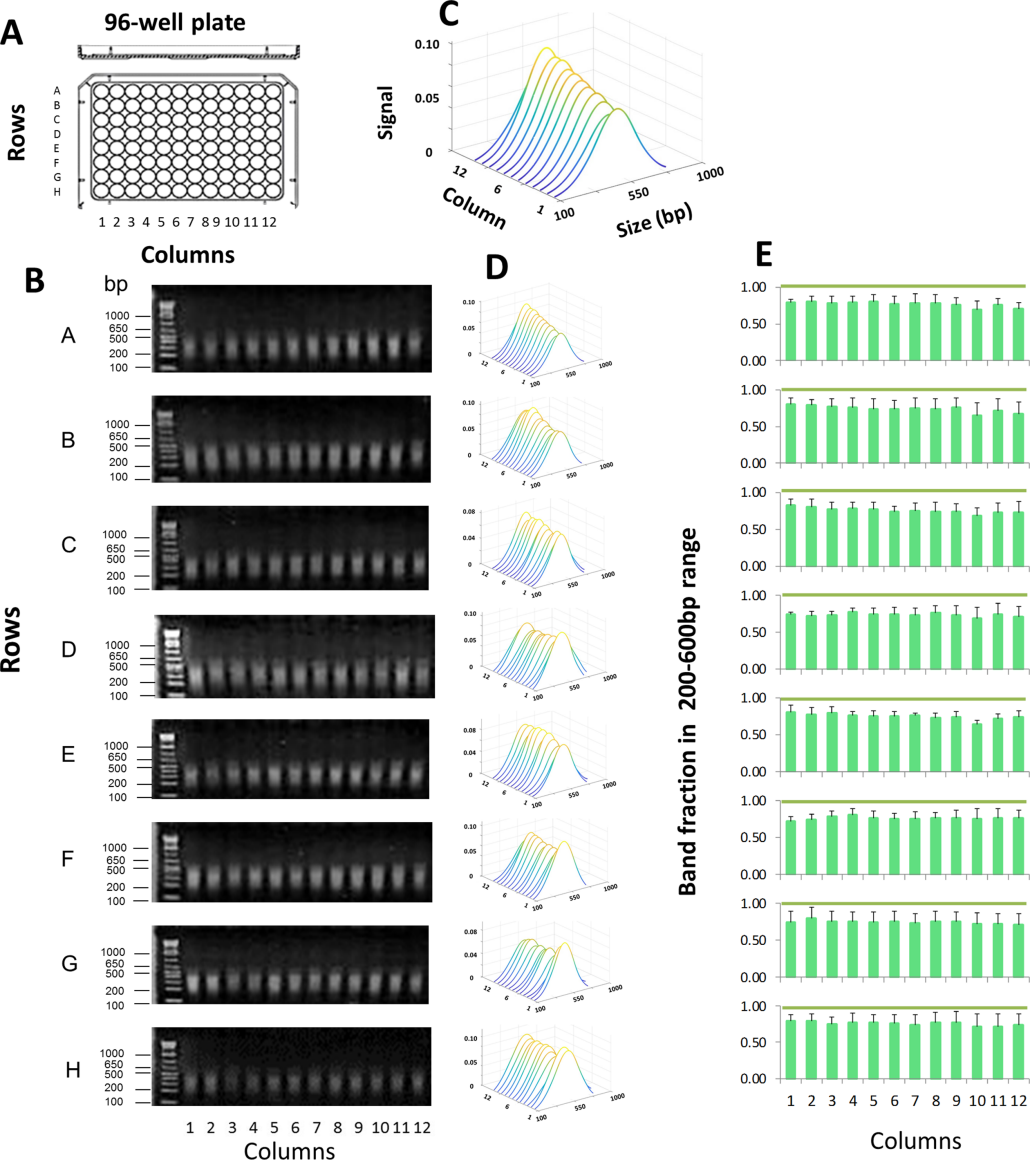
PIXUL shearing of chromatin directly in 96-well plate cell cultures. (**A**) HCT116 cell cultures grown in 96-well plates were crosslinked directly in plates followed by glycine quenching. After PBS wash, shearing buffer was added. Plates were then sealed and were treated in PIXUL (total time 36 min per plate). After digestion with proteinase K and reversal of crosslinking, sheared DNA fragments were resolved by agarose gel electrophoresis. (**B**) Agarose, gels were stained with ethidium bromide. DNA ladder was run in the first lane of each gel. Numbers to the left of the gels show sizes of selected ladder bands in base pair (bp). (**C**) An example to illustrate a waterfall plot (MATLAB) with annotated axis. Image software was used to analyze stained DNA bands (Methods). Results are shown as waterfall plots (MATLAB) of best-fit curves in sequential order of samples from culture plate column well 1 to 12. X- axis; band size in base pair (size (bp)). Y-axis; sample from a well of a given column (*columns 1 through 12*). Z-axis; relative signal intensity of bands for given plate well (Signal). (**D**) Waterfall plots for each plate row (rows A through H). (**D**) Waterfall plots for each plate row. (**E**) Graphs represent band fraction in the 200–600 bp range from each one of the 96 wells (mean ± SDEV, *n* = 4 experiments). These results show that a 96-well plate culture can be directly sonicated with PIXUL, avoiding the sample transfer step and yielding consistent chromatin fragmentation across all 96 wells.

Next, we tested the efficiency of chromatin shearing in HCT116 cells that were cultured and crosslinked in 96-well plates. The cells were washed with PBS in the wells, followed by the addition of shearing buffer. Plates were then sealed and processed using PIXUL. Sheared samples were treated with proteinase K and, after reversal of crosslinking, sizes of DNA fragments were assessed by agarose gel electrophoresis and ethidium bromide staining (Figure 2). Across all wells, the average size of the sheared fragments was 313 ± 56 bp, and on average, 74.7 ± 3.3% of the band fragments were within the 200–600 bp size range (mean ± SDEV, *n* = 4, 96-well culture plates) (Figure 2E).

**Figure 3.**
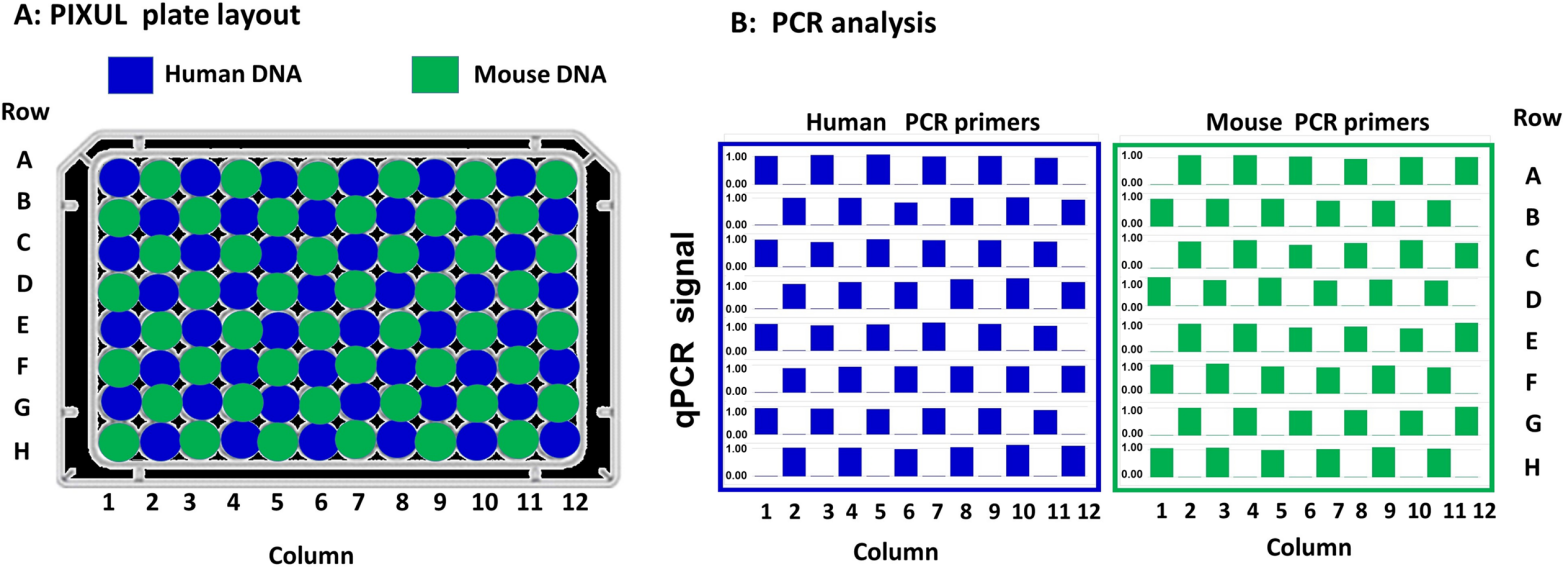
Across 96-well plate contamination test. (**A**) Human and mouse genomic DNA (10 ng/μl in 100 μl volume) were loaded into 96-well plate in a checkerboard layout. After sealing with a film adhesive, plate was treated with PIXUL (18 min per plate), and DNA in each well was assessed using human (*EGR1*) and mouse (*Tnfa*) primers in qPCR. (**B**) Results of qPCR analysis with human (*left panel*) and mouse (*right panel*) primers for each one of the 96-wells (rows A–H and columns 1–12). Bars (*blue* or *green*) in the graphs show relative human and mouse DNA concentrations (each well/average non-zero concentration across the entire plate- scale shown 0.0–1.0). These results demonstrate that there is no detectable (not different from 0.0) cross-contamination across wells.

Although microplates are sealed with adhesive films, there is a concern that during sonication there is cross-contamination between wells. To test for leaks, a 96-well plate was loaded in a checkerboard fashion with either human (‘human wells’, *blue*) or mouse (‘mouse wells’, *green*) genomic DNA (Figure 3A). The sealed plate was treated with PIXUL (18min), and DNA in each well was analyzed in qPCR using either human or mouse primers (Figure 3B). No human DNA was detected in ‘mouse wells’ and no mouse DNA was detected in ‘human wells.’ Thus, these results show that the seal is tight enough to prevent cross-contamination between wells (Figure 3B). We used only one human and one mouse primer. Thus, we might have overlooked contamination that can be detected by more sensitive and general evaluation (e.g. DNA sequencing).

### PIXUL sample preparation combined with Matrix ChIP into an integrated platform for high-throughput chromatin analysis, PIXUL-ChIP

**Figure 4.**
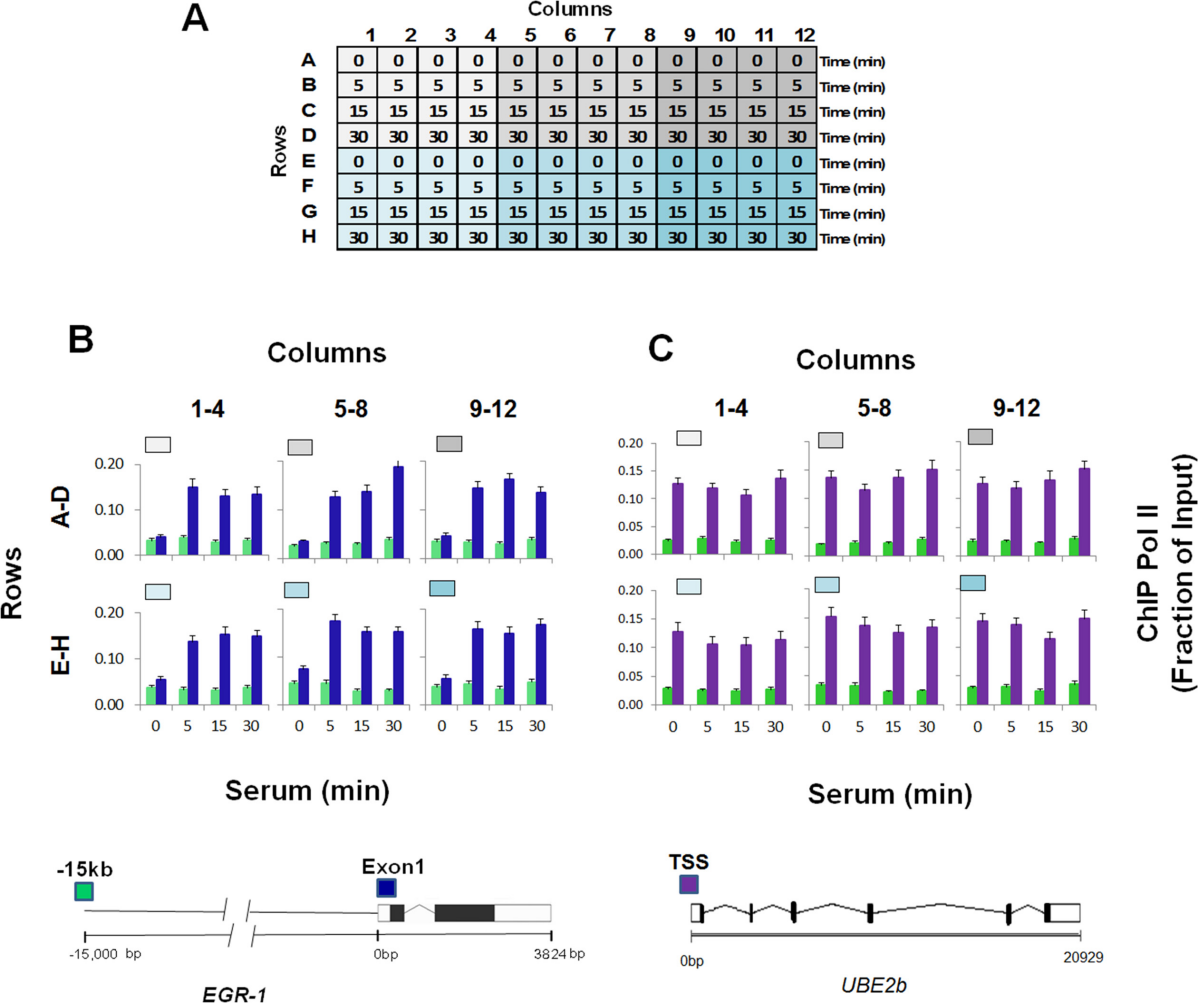
PIXUL-ChIP-qPCR analysis of Pol II recruitment kinetics to the *EGR1* locus in serum-treated 96-well HCT116 culture. Serum-deprived HCT116 96-well cultures were treated with serum for 0, 5, 15 and 30 min. Cells were crosslinked directly in the 96-well plate, quenched with glycine, and washed with PBS. PBS was then replaced with shearing buffer, and the plate was treated with PIXUL. Sheared chromatin was used in Matrix ChIP-qPCR analysis of Pol II at the EGR1 gene. (**A**) Layout of the serum time-course treatment experiment. (**B, C**) Pol II ChIP-qPCR analysis at the *EGR1* (B) and *UBE2b* (C) loci presented as fraction of input. Graphs show mean ± SEM (*n* = 4) of combined ChIP-qPCR as shown (*n* = 4 wells/each time point). Gray/blue boxes above the graphs correspond to colors of the plate quadrants in (A). Cartoons of the *EGR1* and UBE2b genes and location of the PCR primers (colored boxes) are shown below. These results show that cells grown in 96-well plate can be treated with an inducing agent (here, serum) and sonicated directly on the culture plate in PIXUL (no sample transfer), and then sheared chromatin aliquots analyzed in microplate ChIP-qPCR, yielding reproducible results of all 96 samples in one day.

Cell cultures are frequently used to study transcription and epigenetic processes. Many studies are done in 96-well culture plates, which makes the harvesting of cells for epigenetic studies unreliable and tedious. To test the usefulness of PIXUL-generated chromatin in the Matrix ChIP assay, we used a well-characterized model system. We have previously shown that serum added to serum-starved HCT116 cultures activates gene expression and induces recruitment of RNA polymerase II (Pol II) to the *EGR1* locus (25). In ChIP assays, we used Pol II 4H8 monoclonal antibody that recognizes phosphorylated and un-phosphorylated C-terminal domain (CTD) (25,26). This system was utilized to develop a protocol for integrating PIXUL with the Matrix ChIP assay, PIXUL-ChIP. Cells were seeded in 96-well plates in 10% FBS. After reaching near confluence, culture media was replaced with 0.1% FBS to render the culture quiescent. Under these conditions, cells can be maintained quiescent for up to a week, ready for testing when needed. Cell cultures were activated with 10% FBS for 0, 5, 15 and 30 min (Figure 4A). After completion of the time-course serum induction, cells in all 96 wells were cross-linked with formaldehyde, lysis buffer was added, and the plate was treated with ultrasound in PIXUL. 4 μl of sheared chromatin (equivalent to ∼2000 cells) from each well was used in one Matrix ChIP reaction to assess Pol II levels at the inducible *EGR1* and constitutive *UBE2b* loci. The intragenic region 15kb upstream of the *EGR1* gene was used as negative control. The 96 chromatin samples were used in two Matrix ChIP plates (PIXUL rows A–D in Matrix ChIP plate 1 and PIXUL rows E–H in Matrix ChIP plate 2) to run 48 inputs (which is DNA isolated from whole cell extracts) and 48 Pol II ChIPs on each plate. ChIP DNA was assessed by qPCR, and Pol II levels were calculated as a fraction of input as previously described (7). Figure 4A shows the layout of a 96-well culture plate treated with PIXUL. The results demonstrate inducible recruitment of Pol II to the *EGR1* locus, with the kinetics and amplitude similar in each one of the six quadrants (Figure 4B). There were no changes in Pol II recruitment in response to serum at the constitutively expressed *UBE2b* gene, with levels that were similar across all six quadrants, Figure 4C. As expected, Pol II levels were low at the intragenic site 15 kb upstream of the *EGR1* locus (Figure 4B and C).

**Figure 5.**
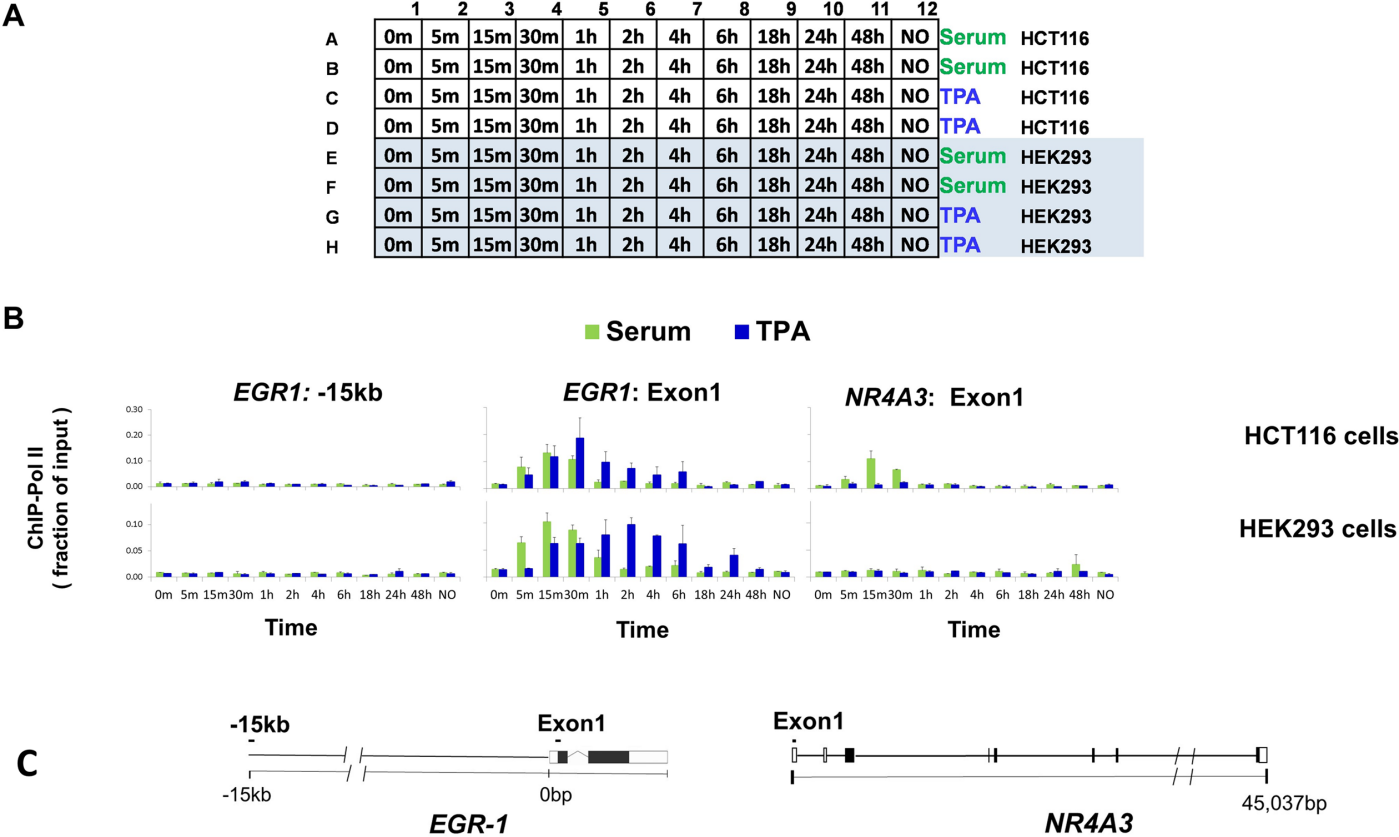
PIXUL-ChIP-qPCR analysis of Pol II kinetics of recruitment to inducible loci in response to serum- and TPA-treatment of 96-well HCT116 and HEK293 cell cultures. Serum-deprived HCT116 and HEK293 cultures in the same 96-well plate were treated with either 10% serum or 100 nM TPA for 5, 15, 30 min and 1, 2, 4, 6, 18, 24 and 48 h. Cells were crosslinked, plates sealed and treated with PIXUL as in Figure 2. Sheared chromatin was used in microplate ChIP analysis of Pol II density at *EGR1* and *NR4A3* genes. (**A**) 96-well plate culture layout of the serum and TPA time-course treatment experiment. (**B**) Graphs of ChIP-qPCR results showing Pol II density (as a fraction of input), mean ± SEM (*n* = 2) of respective cell lines, treatments (serum; *green*, TPA;*blue*) and harvested at indicated time points. (**C**) Gene cartoons and position of PCR primers. These data show that different cells can be cultured on the same 96-well plate, treated with different agents at various times, and then sheared directly in PIXUL and analyzed by ChIP-qPCR yielding results for all 96 samples in one day.

Next, we compared side-by-side different cell types grown on the same plate and treated with different agents over a time-course. Two human lines, HCT116 and HEK293, were grown on the same 96-well plate. After serum deprivation, the quiescent cells (columns 1–11) were treated with either serum or TPA over a time course from 0 min to 48 h prior to crosslinking. Included was also a set of wells in which cells were maintained in 10% serum without any treatment (column 12). To assess the reproducibility of the entire experiment, treatments were done in duplicates. The layout of the plate for this experiment is shown in Figure 5A. The results of ChIP analysis show that both serum and TPA increased levels of Pol II at the *EGR1* gene in HTC116 and HEK293 cells but that the kinetics of induction were different. Further, only HCT116 cells demonstrated serum-inducible Pol II recruitment to the *NR4A3* locus (27).

These results show that PIXUL sample preparation can be easily integrated with downstream microplate ChIP assays, providing a useful tool that facilitates ChIP studies where comparative analyses of a number of cell lines and/or treatments are done in parallel (28). Further, starting with a 96-well culture plate, sample preparation and all steps of the ChIP assay and qPCR analysis are completed in the same day. As such, along with other applications, integrated PIXUL-ChIP should be a useful tool for drug screening and validation.

### PIXUL-ChIP application to embryonic stem cells (ESC)

**Figure 6.**
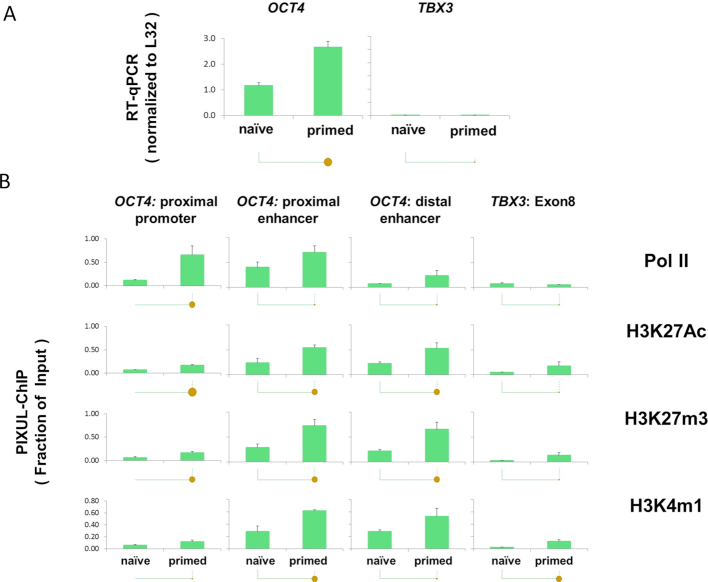
PIXUL-ChIP-qPCR analysis of Pol II and epigenetic modifications at the *OCT4 (POU5F1)* locus in hESC Elf1 cells. Human embryonic stem cells (hESC Elf1) were cultured in 96-well plates as naive (2i + hLif + FGF2 + Igf1) or as primed (TeSR+FGF2) on Matrigel for either one or two passages. (2i- two inhibitors: PD0325901 MEKi and CHIR-99021GSK3i). One and two passages in TeSR represent cells transitioning to primed. These cells were plated at 10 000 cells/well on Matrigel in 96-well plates with Rho kinase (ROCK) inhibitor present for the first 24 h of culture to improve survival (32). Half of the plate was used to extract RNA for RT-qPCR (normalized to L32 mRNA) (**A**) and the other half (chromatin) was crosslinked, sheared in PIXUL, and subjected to Matrix ChIP analysis (expressed as a fraction of input) (**B**) as in Figure 3. Statistical differences between two means (*P* value) are shown by the size of the solid circles: *P* < 0.05 for small circle, *P* < 0.01 for large circle, and no circle indicating the differences are not statistically significant (7). These results are consistent with previous observations and as such demonstrated that PIXUL-ChIP-qPCR platform could be used for high-throughput experiments and drug screening.

A number of small molecules, including epigenetic drugs, have been discovered to induce pluripotency (29) and manipulate ESC fate (30,31). Still, these studies are limited by the lack of sensitive technologies that would allow high-throughput testing and validation of drugs in ESCs. To test PIXUL-ChIP applicability in ESCs, we used Elf1 hESC derived from blastocysts of frozen 6–8-cell embryos (NIHhESC-12-0156) (32,33). 96-well plates with either naïve (Elf1 2iLIF) or primed (Elf1, 2 passages in TeSR + FGF2 media for 4 days) cells were set up (32,34,35) for PIXUL-ChIP and RT-qPCR analysis. After cells in some of the wells were harvested for RT-qPCR, the rest of the plate was cross-linked and sonicated with PIXUL (24min). Sheared chromatin was used in Matrix-ChIP-qPCR analysis as before (Figures 4 and 5). High levels of expression and high chromatin accessibility of the *OCT4 (POU5F1)* locus is a hallmark of ESC, including Elf1 cells (32). RT-qPCR demonstrated high levels of *OCT4* expression, which was higher in TeSR+ FGF2-primed cells compared to 2iLIF naïve cells (Figure 6A). In contrast, expression of another transcriptional regulator, *TBX3*, (36) was very low in both cells. PIXUL-ChIP analysis (Figure 6B) demonstrated high levels of permissive (H3K27Ac and H3K4m1) (37) and repressive (H3K27m3) epigenetics marks at the *OCT4* enhancers compared to promoter regions, and these modifications were higher in the primed cells compared to naïve cells. Consistent with the mRNA data (Figure 6A), Pol II levels and marks were low at the *TBX3* gene. These observations are consistent with previous observations that in primed Elf1 cells, *OCT4* enhancers have higher chromatin accessibility (DNase I hypersensitivity) and H3K27me3 levels compared to naïve cells (32).

These studies illustrate that PIXUL-ChIP is a tool that has the potential to empower researchers for high-throughput screens (such as small molecules and growth factors) to study ESC self-renewal and pluripotency more readily than the traditional approach.

### Comparison of integrated PIXUL-ChIP protocol with commercial Bioruptor and Covaris chromatin shearing instruments followed by Matrix ChIP

There are several commercially available ultrasound instruments to sonicate chromatin. The two best known are the Bioruptor (manufactured by Diagenode), and LE220 Focused Ultrasonicator (manufactured by Covaris).

#### Bioruptor

**Figure 7.**
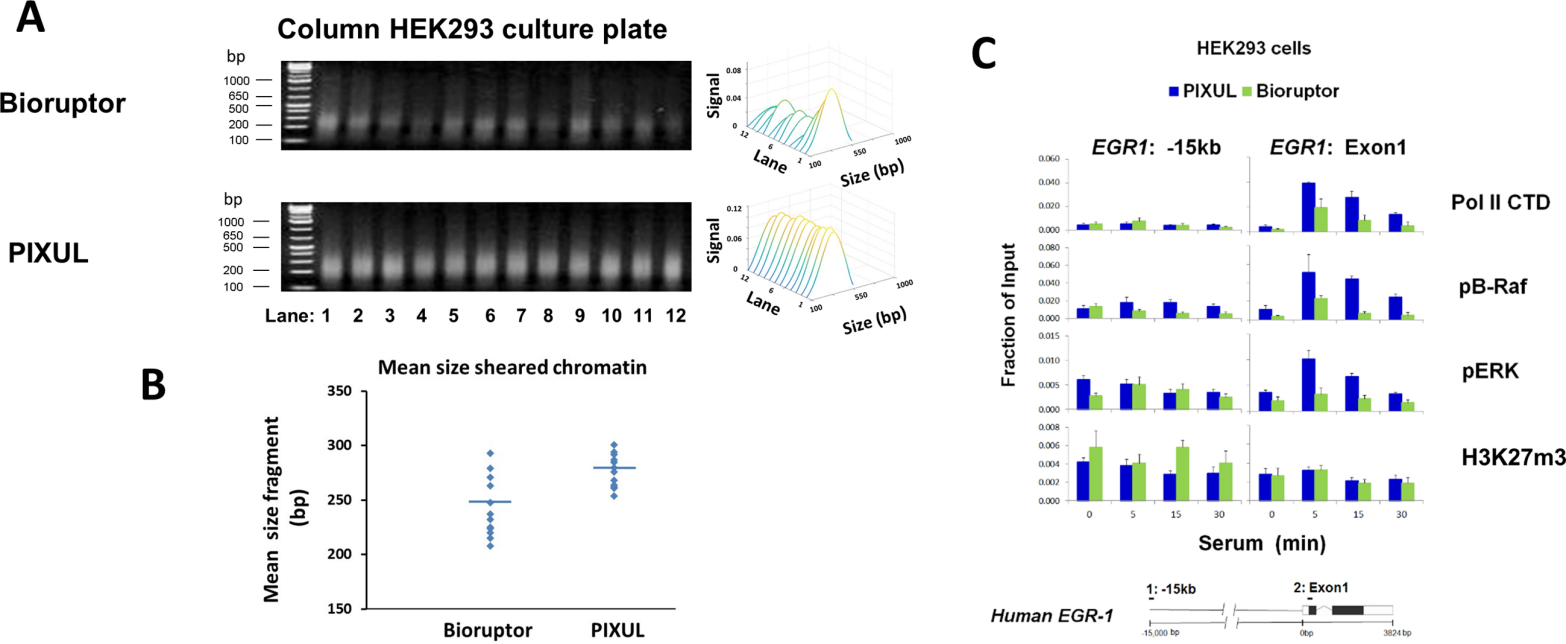
Matrix ChIP analysis of chromatin prepared from 96-well HEK293 cultures using PIXUL and Bioruptor. Serum-deprived HEK293 96-well cultures were treated with serum for 0, 5, 15 and 30 min. Cells were crosslinked directly in the 96-well plate, harvested manually from Row A (12 samples) and transferred to twelve 0.5 ml tubes for ultrasound treatment in the Bioruptor (45 min). The rest of the plate was sealed and sonicated in PIXUL (26 min treatment). (**A**) comparison of sheared chromatin fragments obtained with Bioruptor versus PIXUL analyzed by agarose gel electrophoresis, Ethidium bromide stained gels are shown, sizes (bp) of DNA ladder fragments (first lane) are shown to the left. Sonicated fragments were analyzed by image analysis software (Methods), results are displayed as waterfall plots in sequential order of samples run from lane 1 to 12. X- axis; band size in base pair (bp). Y-axis; sample from a given lane. Z-axis; relative signal intensity of bands. (**B**) Mean fragment size (each blue dot) of sheared chromatin obtained with Bioruptor and PIXUL. (**C**) Sheared chromatin samples from both PIXUL and Bioruptor were analyzed simultaneously by Matrix ChIP using antibodies to Pol II, pB-Raf, pErk and H3K27m3. ChIPed DNA was analyzed by real-time PCR using indicated primers. Results show mean ± SEM (*n* = 3 replicates for each time point for each instrument). This comparison demonstrates that sample transfer causes significant sample losses, which may in part account for greater variability. There are lower Matrix ChIP signals from chromatin prepared with Bioruptor compared to PIXUL.

Bioruptor uses standard test tubes and can process 12 tubes at a time. We compared the efficiency of PIXUL-ChIP with Bioruptor followed by Matrix ChIP. Quiescent HEK293 cells in a 96-well plate were treated with serum (0, 5, 15 and 30 min). Next, one row of cells (12 wells, *n* = 3 for each time point) was transferred to test tubes, crosslinked and then sheared in the Bioruptor (45 min sonication). The rest of the plate was crosslinked and treated with PIXUL (36 min sonication). Agarose gel electrophoresis (Figure 7A) shows less chromatin yield using the Bioruptor protocol compared to PIXUL, suggesting that losses were associated with manual harvesting of the cells from 96-well plates and transfer to tubes for sonication in the Bioruptor. Lower yields of Bioruptor-sheared chromatin are also illustrated for HCT116 cells in Supplementary Figure S3. The average size of HEK293 cell Bioruptor-sheared fragments was 243 ± 28 bp (77.2 ± 7.3% in 200–600 bp range), comparable to 277 ± 15 bp (84.3 ± 2.5% in 200–600 bp range) with PIXUL (mean ± SDEV, *n* = 12 wells/samples). This comparison shows that chromatin fragmentation with PIXUL done directly in culture plates is more consistent compared to fragmentation with Bioruptor done in tubes requiring sample transfers (Figure 7B). Activation of *EGR1* gene is associated with recruitment of active (phosphorylated) components of the ERK pathway to the *EGR1* locus (25,26,38). Equal aliquots of chromatin from PIXUL- and Bioruptor-generated samples were analyzed on the same Matrix ChIP plate using antibodies to Pol II CTD, H3K27m3, B-Raf phosphorylated on T598 and S601 (pB-Raf) and ERK phosphorylated on T202 and Y204 (pERK) (25,26). As before, serum treatment increased Pol II recruitment to the *EGR1* gene, but the measured levels were higher in chromatin samples prepared with PIXUL compared to Bioruptor (Figure 7C). Measured levels of serum-induced pB-Raf and pERK at *EGR1* gene were also higher in chromatin samples prepared with PIXUL. In contrast, levels of H3K27m3 were similar using the PIXUL and Bioruptor. Pol II CTD, pB-Raf and pERK antibodies recognize phosphorylated forms of these proteins (25,26). Phosphorylation can be significantly degraded during sample preparation prior to analysis (7,39), which could explain the differences between the two methods (where Bioruptor protocol requires more manual handling and longer preparation times).

We also tested the efficiency of PIXUL versus Bioruptor in chromatin sample preparation from approximately similar size pieces of frozen livers from a mouse model of sepsis. Sheared liver chromatin yields were similar with both methods (Supplementary Figure S4A), providing further evidence that the differences seen with cell cultures (Figure 7A) occur during sample harvest and transfer from the 96-well plate to Bioruptor tubes. The average size of Bioruptor-sheared fragments was 258 ± 14 bp compared to 362 ± 10 bp with PIXUL (mean ± SDEV, *n* = 6 livers). Previously we found that in experimental sepsis models there was an increased recruitment of Pol II to *Ngal* (*Lcn2*) in liver (40). Both sonication methods showed an increase in Pol II signal at the *Ngal* (*Lcn2*) gene in septic livers, but the level was greater in chromatin prepared by PIXUL (Supplementary Figure S4D) compared to Bioruptor. As a no-change control, we assessed H3 levels, which were not altered by sepsis but were again higher in chromatin prepared with PIXUL compared to Bioruptor.

Our Matrix ChIP results show that PIXUL that uses microplates is faster and more efficient than the standard tube-based Bioruptor approach, where loss of samples during manual transfers and partial dephosphorylation may underlie lower Pol II CTD, pB-Raf and pERK levels at genes.

#### Covaris LE220

**Figure 8.**
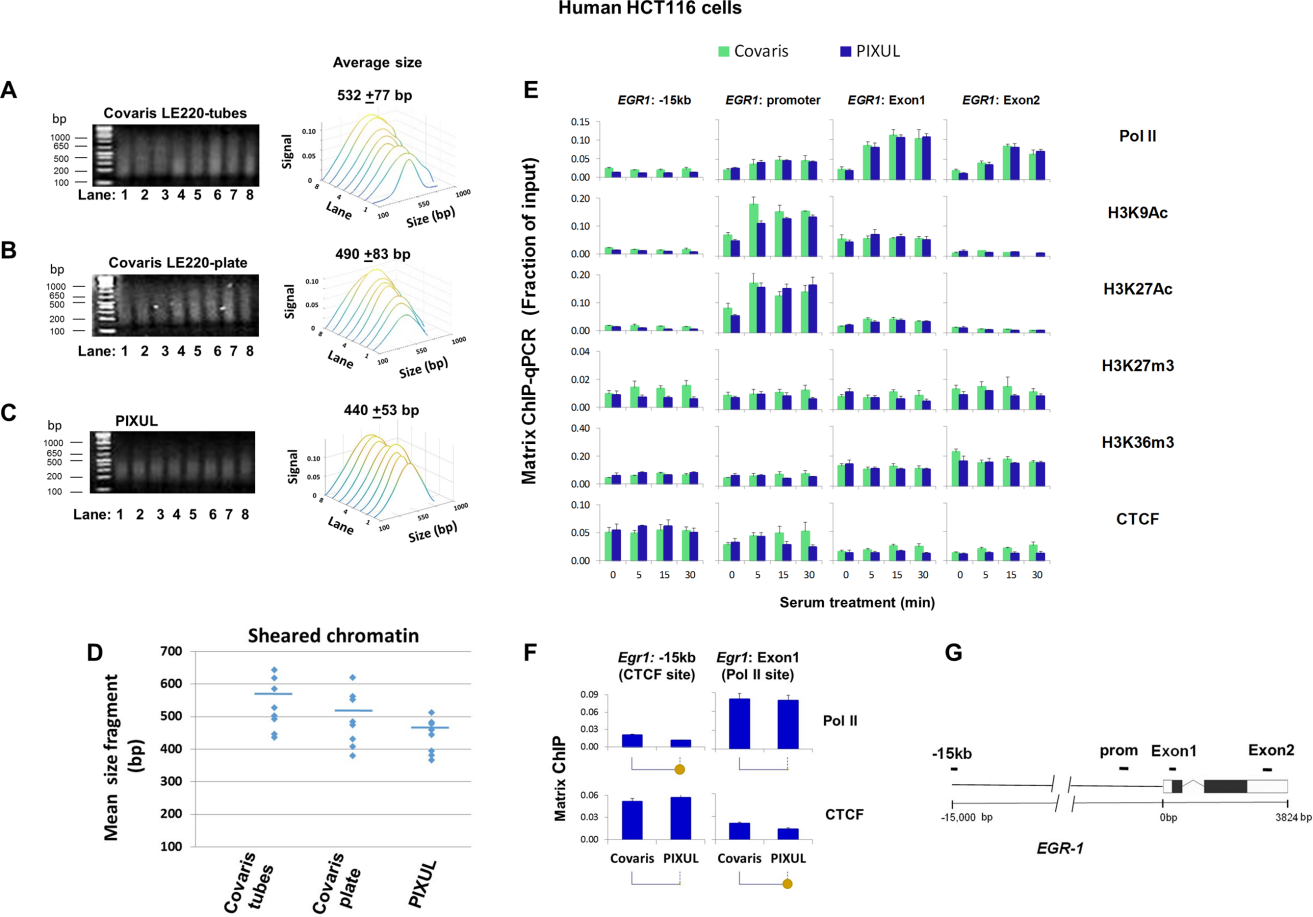
Matrix ChIP analysis of chromatin prepared from 96-well HCT116 cultures using either PIXUL or Covaris LE220. Serum-deprived HCT116 cell 96-well plate cultures were treated with serum for 0, 5, 15 and 30 min. Cells were cross-linked directly in the 96-well plate. With Covaris LE220 shearing harvesting cells from one well of a 96-well plate yielded insufficient amounts of chromatin to generate reproducible ChIP results. Thus, with this instrument, for each time point cells harvested from three wells of a 96-well plate were combined into one sample and transferred to either Covaris microplate tubes or Covaris microplate. Each time point was done in duplicate, for a total of eight samples. The rest of the 96-well plate was sealed and sonicated in PIXUL (18 min treatment). (A–C) Agarose gel electrophoresis analysis of chromatin fragments sonicated using Covaris LE220 microplate tubes (**A**), Covaris LE220 plate (**B**) or PIXUL (**C**). Sheared fragments were analyzed by image analysis software (Methods), results are displayed as waterfall plots in sequential order of samples run from lane 1 to 8 as in Figure 1. X- axis; band size in base pair (bp). Y-axis; sample from a given lane. Z-axis; relative signal intensity of bands. Numbers above the plots show average fragment size ±SEM for all eight samples. (**D**) Sizes of chromatin samples (A-C) sonicated by either Covaris tubes, Covaris plate or PIXUL. (**E**) Sheared chromatin samples prepared using either Covaris tubes (cells from 3 wells combined into one sample) and PIXUL (single well per sample) were analyzed simultaneously by Matrix ChIP using antibodies to Pol II, H3K9Ac, H3K27Ac, H3K27m3, H3K36m3 and CTCF. ChIP DNA was analyzed by qPCR using indicated primers. Results show fraction of input, mean±SEM (*n* = 3 replicates for each time point for each instrument). (**F**) Comparison of Pol II and CTCF ChIP signals at known binding and respective distal sites using Covaris versus PIXUL sonicated chromatin (yellow circles below graphs show *P* < 0.05). (**G**) *EGR1* gene cartoon and position of PCR primers. This comparison demonstrates that sonication of chromatin with PIXUL is more consistent and yields smaller fragments compared to Covaris; in particular the first position (A1) in their plate yields considerably smaller fragment than the other wells. Combing cells from three wells of a 96-well plate for Covaris sonication generates chromatin yielding similar ChIP results to those obtained using cells from one well of a 96-well plate treated with PIXUL. The ChIP background signal is lower with PIXUL compared to Covaris.

This instrument uses either glass tubes or glass microplates. The ultrasound transducers and the plates/tubes are physically moved during the operation, and the water used to couple ultrasound to wells requires degassing. With PIXUL, neither the transducers nor the plate move, and no degassing of the coupling fluid is performed. To compare Covaris with PIXUL, we used the serum-treated HCT116 cell culture system as above (Figure 4). We found that harvesting cells from one well of a 96-well culture plate yielded insufficient amounts of chromatin in Covaris to generate reproducible ChIP data. Thus, for Covaris LE220 we combined cells from three wells of a 96-well culture plate into one sample. The sizes of chromatin fragments sonicated with Covaris were not uniform (Figure 8A and B). Notably, the first position/well (A1) of the Covaris sonicator yielded smaller fragments with either tubes or plate (Figure 8A and B, lane 1). The mean fragment size was 532±77 for Covaris tubes and 490 ± 83 for Covaris microplate, compared to 440 ± 53 for PIXUL (Figure 8A–D). Chromatin prepared with either Covaris or PIXUL instruments and tested in Matrix ChIP yielded similar signals (Figure 8E), but the background was lower using PIXUL (Figure 8F). This comparison demonstrates that PIXUL, which uses inexpensive off-the-shelf plates, not only avoids manual transfers from 96-well culture plates (allowing the use of lower cell numbers) but also shears chromatin more consistently compared to the Covaris LE220 instrument (Figure 8A–D, and also see Figure 2 for all 96 wells).

Covaris instruments are widely used for genomic applications. We thus compared human genomic DNA shearing using PIXUL to Covaris LE220 for exome sequencing library preparation (Supplementary Figure S5). As shown, the quality of exome sequencing libraries was similar with both instruments. Thus at much lower operating costs, PIXUL can also be used as a sample preparation platform for genomic applications.

### PIXUL-ChIP analysis of Pol II occupancy at organ-restricted genes in mouse heart, kidney, liver, and lung

**Figure 9.**
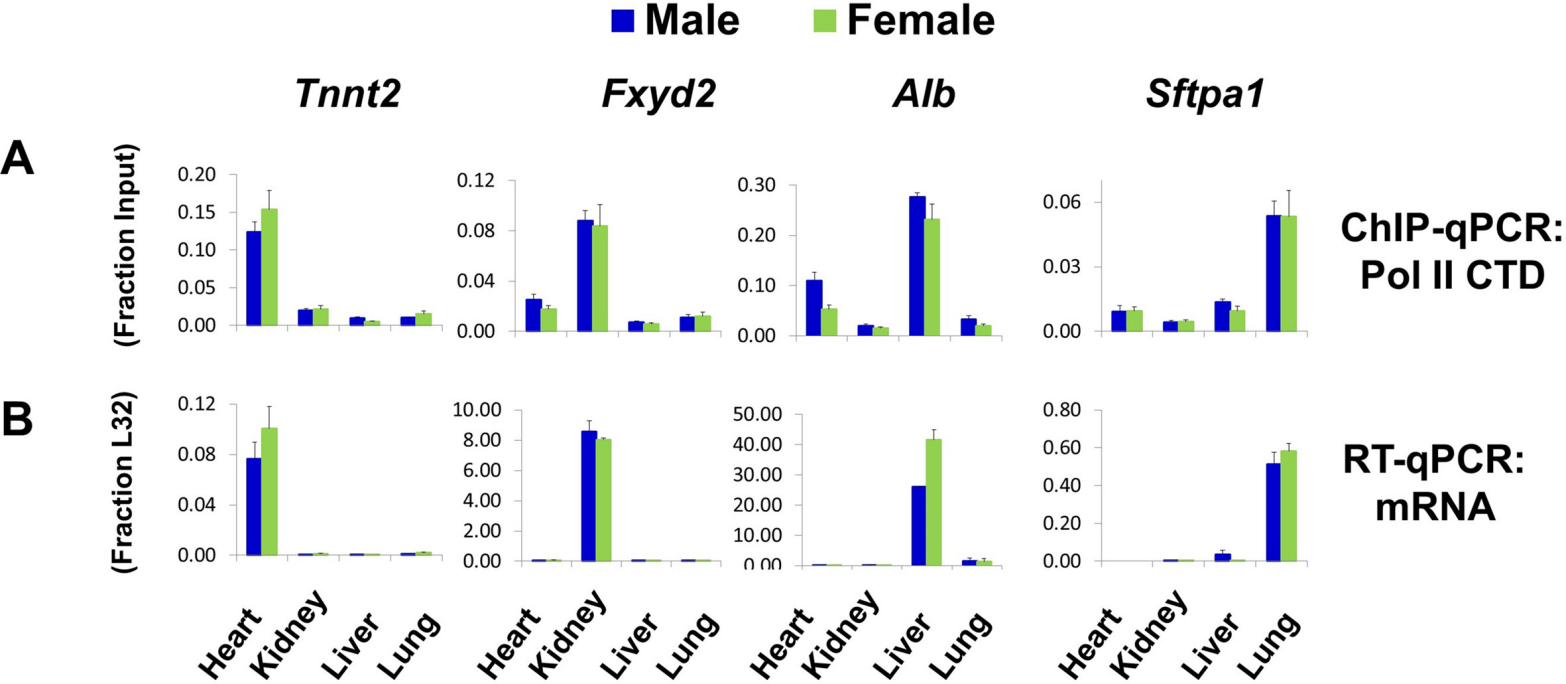
PIXUL-ChIP analysis of Pol II occupancy in mouse heart, kidney, liver, and lung. Flash frozen heart, kidney, liver and lung samples from male and female mice were cross-linked and then sonicated in microplates using PIXUL. (**A**) PIXUL-sheared chromatin samples were simultaneously analyzed for Pol II levels at indicated organ-specific genes using Matrix ChIP. ChIP DNA was analyzed by qPCR expressed as fraction of input. Data represent mean ± SEM (*n* = 3 mice) expressed as a fraction of input. (**B**) RNA isolated from the same frozen organs as in A was used in RT-qPCR with primers to indicated genes. Data represent mean ± SEM (*n* = 3 mice) expressed as a ratio to the transcript levels of housekeeping ribosomal protein gene, L32. These results demonstrate that PIXUL-ChIP can be used to analyze multiple samples from several organs on the same plate.

As many diseases are associated with systemic epigenetic changes (e.g. diabetes, obesity, inflammation, sepsis and even cancer), having methods for parallel multiple organ studies in model systems would offer new potential to better understand epigenetics of disease progression and evaluate drug efficacy/toxicity in different organs, ultimately providing information to improve clinical outcomes (40–42). We harvested hearts, kidneys, livers and lungs from male and female mice and simultaneously prepared chromatin samples from fragments of all these tissues in a single 96-well plate using PIXUL. Figure 9A illustrates Pol II binding to genes known to be preferentially expressed in the heart, *Tnnt2* (troponin); kidney, *Fxyd2* (ATPase subunit); liver, *Alb* (albumin); and lung, *Sftpa1* (surfactant). The organ-specific Pol II binding was corroborated by RT-qPCR measurements of cognate transcripts (Figure 9B). The above experiment demonstrates that PIXUL integrated with Matrix ChIP facilitates parallel high-throughput epigenetic analysis of multiple organs.

The novel ultrasound transducer design, the use of off-the-shelf inexpensive plates, and user-friendly operation give PIXUL the potential to be used as a multipurpose sample preparation platform (e.g. in integrative studies). To test this concept, we show that PIXUL can be used for multiorgan RNA isolation done in parallel with chromatin shearing, for RT-PCR and ChIP assays (Supplementary Figure S6).

### PIXUL-ChIP-seq

ChIP-seq is a widely used method that provides powerful means to assess histone modifications and chromatin-bound proteins genome-wide (18,43–45). Sonication is commonly used to shear chromatin for ChIP-seq. We assessed the compatibility of PIXUL with an established ChIP-seq pipeline (Active Motif). HCT116 cells cultured in 96-well plates (∼200 000 cells/well) were sonicated in PIXUL as above, ChIP was carried out with different antibodies, and libraries were constructed and sequenced (see Methods).

**Figure 10.**
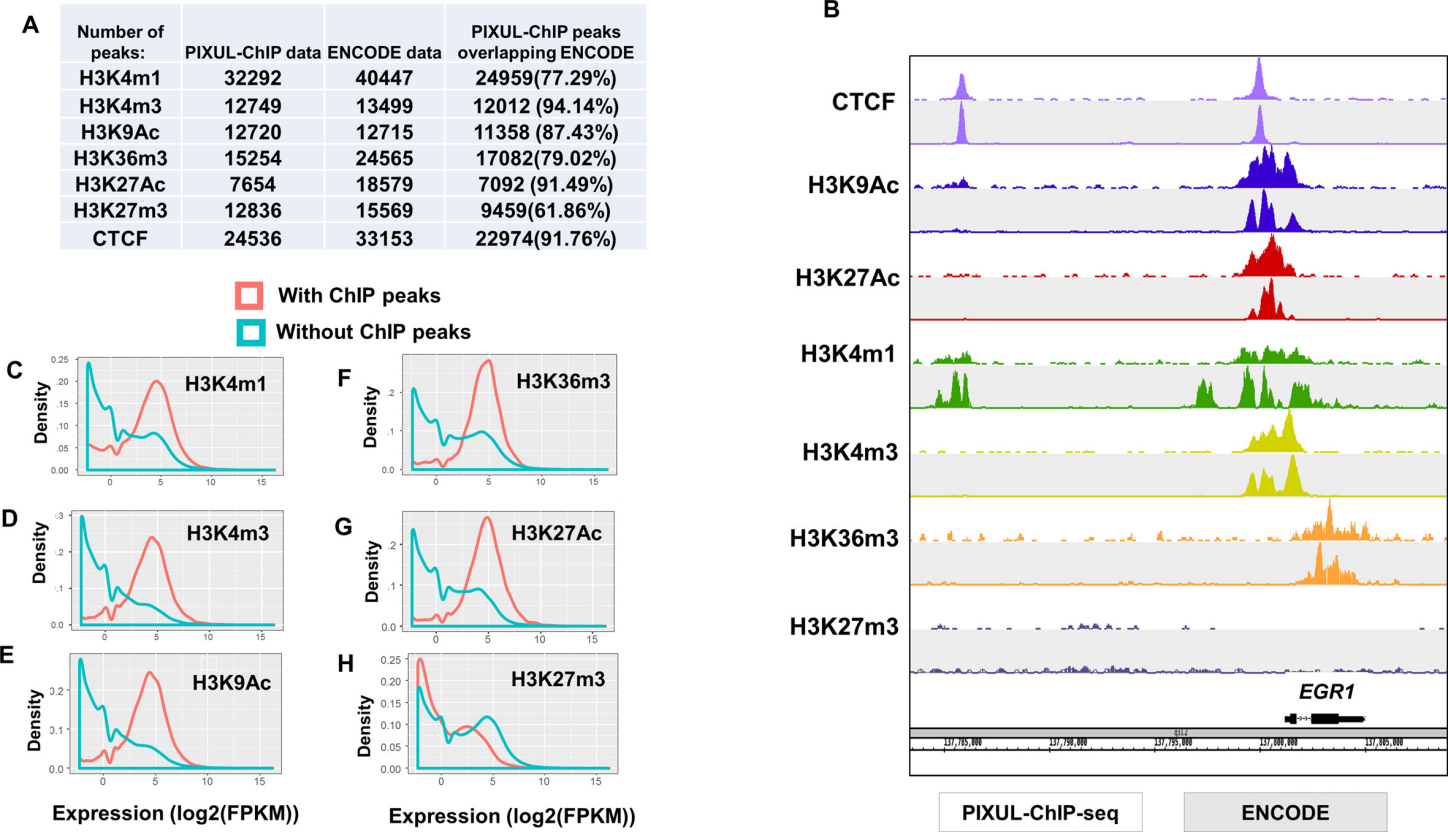
PIXUL-ChIP-seq results and comparison to ENCODE datasets. HCT116 cells were grown to the density of ∼200 000 cells per well, cross-linked, and sonicated using 96-well PIXUL. ChIP was performed and libraries were generated from a single PIXUL well using Active Motif's Low Cell ChIP-Seq Kit. Libraries were sequenced on a NextSeq 500 (Methods). (**A**) Number of peaks in PIXUL-ChIP-seq and ENCODE datasets, and percentage of PIXUL-ChIP-seq peaks that are also detected in ENCODE. (**B**) PIXUL-ChIP-seq (*white background*) and ENCODE (*gray background)* genome browser snapshot of a region around the *EGR1* locus occupied by CTCF, H3K9Ac, H3K27Ac, H3K4m1, H3K4m3, H3K36m3 and H3K27m3. The data demonstrate good agreement between PIXUL-ChIP-seq (which was done in ∼200,000 cells) compared to ENCODE (which used >10^6^ cells). (C–H) To verify that genes marked by PIXUL-ChIP-seq peaks show expected expression patterns (genes with repressive marks have lower expression, genes with active marks show higher expression), HCT116 RNA-seq data were downloaded from Sanger Institute Genomics of Drug Sensitivity in Cancer (GDSC) website (https://www.cancerrxgene.org/). Expression distribution was plotted of genes with histone marks at the transcription start sites (TSS) and those without. Genes marked with active histone marks around TSS have a mean expression of 32 (2^5^) Fragments Per Kilobase Million (FPKM), while genes without active histone marks (or with repressive mark H3K27m3) are expressed < 1FPKM – resulting in the bimodal distribution. (**C**) Expression distribution for genes with H3K4m1 PIXUL-ChIP-seq peaks (orange) around TSS and genes without H3K4m1 peaks (blue). (**D**) Expression distribution for genes with H3K4m3 peaks around TSS and genes without H3K4m3 peaks. (**E**) Expression distribution for genes with H3K9Ac peaks around TSS and genes without H3K9Ac peaks. (**F**) Expression distribution for genes with H3K36m3 peaks around TSS and genes without H3K36m3 peaks. (**G**) Expression distribution for genes with H3K27Ac peaks within gene body or around TSS and genes without H3K27Ac peaks. (**H**) Expression distribution for genes with H3K27m3 peaks within gene body or around TSS and genes without H3K27me3 peaks.

We compared 7 ChIP-seq HCT116 cell signals (H3K4m1, H3K4m3, H3K9Ac, H3K36m3, H3K27Ac, H3k27m3 and CTCF) that are profiled by both PIXUL ChIP and the ENCODE project (Figure 10). We found that the majority (62–94%) of peaks identified in our PIXUL-ChIP samples are also identified as peaks in the corresponding ENCODE samples (Figure 10A). Figure 10B illustrates a snapshot at the *EGR-1* locus comparing PIXUL-ChIP-seq and ENCODE (for a link to UCSC Genome Browser track, see Methods). Scatter plot (46) analysis for all of the above antibodies demonstrated good correlation between PIXUL-ChIP-seq and ENCODE datasets (Supplementary Figure S7). The differences between PIXUL-ChIP-seq and ENCODE data sets may reflect the use of ChIP antibodies from different sources, growth conditions, and the lower number of HCT116 cells (∼200 000 for PIXUL-ChIP-seq) compared to ENCODE (>10^6^).

To verify that genes marked by PIXUL-ChIP-seq peaks show the expected expression pattern (genes with repressive marks have lower expression, genes with active marks have higher expression), HCT116 RNA-seq data were downloaded from the Sanger Institute Genomics of Drug Sensitivity in Cancer (GDSC) website (https://www.cancerrxgene.org/). Plots of expression distributions of genes showed expected correlations with histone marks (H3K4m1, H3K4m3, H3K9Ac, H3K27Ac, H3K27m3, H3K36m3) at the transcription start sites (TSS) (Figure 10C–H).

In summary, we have developed an ultrasound instrument, PIXUL, to rapidly sonicate chromatin and DNA in standard 96-well tissue culture plates and integrated it with ChIP for high-throughput ChIP-qPCR and ChIP-seq analysis. The integrated PIXUL-ChIP method has several important advantages over existing protocols. (i) 96-well plates with cell cultures are directly placed in PIXUL so that cell harvesting and sonication is done in one step, limiting losses during sample transfers (Figures 4–6). Other sonicators use tubes or 96-well glass plates, requiring manual transfers and inherently resulting in sample losses. With PIXUL, fewer transfer steps potentially minimize epitope losses (Figure 7). (ii) PIXUL high-throughput chromatin shearing in microplates matches the format and throughput of the microplate ChIP platform (Figures 4 and 5) (8,9,11). This feature allows for efficient integration of sample preparation with downstream analytical steps, with the potential to fully automate the entire process from sample preparation to results. (iii) ChIP studies involving multiple cell lines and treatments can be carried out on the same 96-well culture plate, making it well suited for high-throughput kinetic studies or drug screening experiments (Figure 5). (iv) Dozens of tissue samples can be processed in parallel. This feature might be useful, for example, in multiple organ (Figure 9, Supplementary Figure S6) or intratumor epigenetic heterogeneity studies. (v) PIXUL can be used in genome-wide sequencing studies (Figure 10 and Supplementary Figure S5). (vi) PIXUL has the potential as a multipurpose and multiomics sample preparation platform (Figure 10, Supplementary Figure S5-S6). (vii) PIXUL consumables cost a small fraction of expenses associated with use of other comparable systems (such as Covaris). The substantial cost reductions allow for more labs to carry out high-throughput studies.

## DATA AVAILABILITY

Sequencing data was deposited in Gene Expression Omnibus database under entry GSE115822.

## Supplementary Material

gkz222_Supplemental_File
