# Supplementary material for: PIXUL-ChIP: integrated high-throughput sample preparation and analytical platform for epigenetic studies
Source: Nucleic Acids Res. 2019 Mar 30;47(12):e69. doi: 10.1093/nar/gkz222 (PMC6614803; doi:10.1093/nar/gkz222)
Supplement: gkz222_Supplemental_File [file gkz222_supplemental_file.pdf]

Supplementary information

NAR-01982-Met-K-2018R3— (gkz222).

PIXUL-ChIP: high throughput sample preparation and analytical platform for epigenetic studies

Karol Bomsztyk[1,2*], Daniel Mar[1], [1, 2, 3]Yuliang Wang, Oleg Denisenko[1], Carol Ware[2], Christian D. Frazar[4], Adam Blattler[5], Adam D. Maxwell[6,7], Brian E. MacConaghy[7], Thomas J. Matula[7]

[1]UW Medicine South Lake Union, University of Washington, Seattle, WA 98109, USA, [2]Institute for Stem Cell and Regenerative Medicine, University of Washington, Seattle, WA 98109, USA, [3]Paul G. Allen School of Computer Science &Engineering, University of Washington, Seattle, WA 98195, USA, [4]Department of Genome Sciences, University of Washington, Seattle, WA 98195, USA, [5]Active Motif, Carlsbad, CA, 92008, USA [6]Department of Urology, University of Washington School of Medicine, Seattle, WA 98195 and [7] Center for Industrial and Medical Ultrasound, Applied Physics Laboratory, University of Washington, Seattle, WA 98195,

*Address for correspondence:
Karol Bomsztyk
UW Medicine at Lake Union
Box 358050
University of Washington,
Seattle, WA 98109 USA
Tel. (206) 616-7949
E-mail: karolb@u.washington.edu

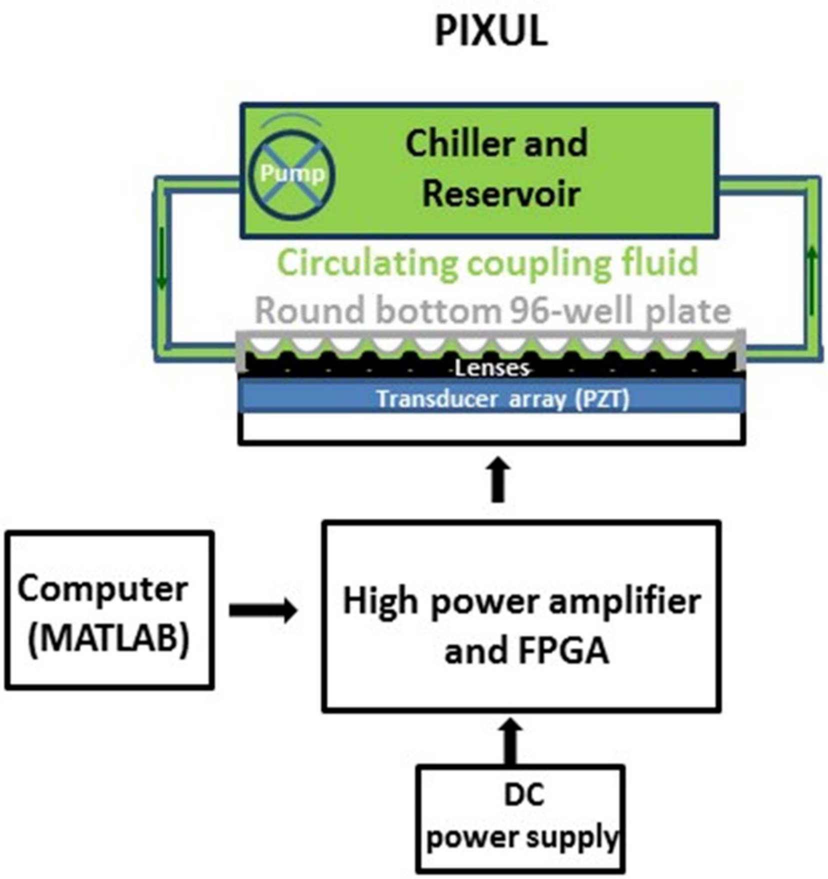

Fig.S1. Schematic diagram of PIXUL. PIXUL is in house-built system comprised of the following main units: (1) a transducer-lens assembly capable of focusing ultrasound in each well of a 96 well microplate, (2) a high power amplifier to drive the transducer array, (3) a chiller- reservoir-pump that circulates ultrasound coupling fluid to reduce heating of the samples, and (4) a computer to control (MATLAB) the ultrasound pulse parameters (number of cycles, treatment configurations, and treatment time).

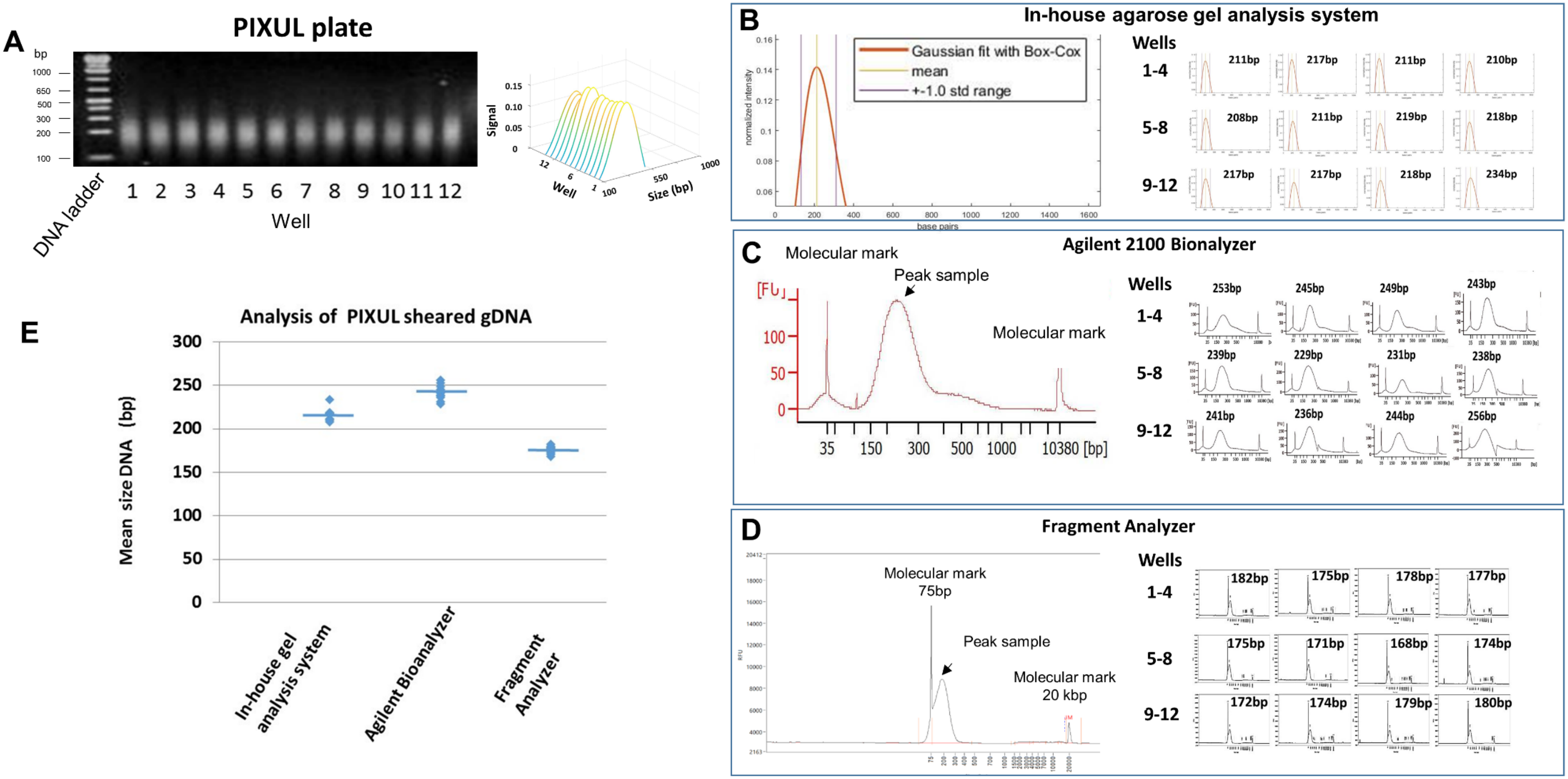

F

Well		1	2	3	4	5	6	7	8	9	10	11	12	Average	SDEV
In-house gel analysis system	(size bp)	211	217	211	210	208	211	219	218	217	217	218	234	215.9	6.9
Agilent 2100 Bioanalyzer	(size bp)	253	245	249	243	239	229	231	238	241	236	244	256	242.0	8.2

Fig. S2. Analysis of PIXUL sheared genomic DNA bands using in-house agarose gel electrophoresis system, Agilent 2100 High Sensitivity DNA Bioanalyzer DNA and Fragment Analyzer. gDNA from A row of 12 wells of 96-well plate was analyzed using either in-house agarose gel electrophoresis/ MATLAB image analysis tool, Agilent High Sensitivity Agilent 2100 Bioanalyzer (Agilent) or Fragment Analyzer (Agilent). ***A***, Ethidium bromide stained agarose gel after electrophoresis of sheared gDNA. Numbers to the left of the gels show sizes of selected ladder bands in base pair (bp). Sheared fragments were analyzed by agarose gel electrophoresis image software (Methods) and shown here as waterfall plots (right panel) that contain best-fit curves of samples 1 to 12 in sequential order. X- axis; band size in base pair (bp). Y-axis; sample from a given well. Z-axis; relative signal intensity of bands for given plate column. ***B***, Plots of DNA bands from 12 wells analyzed with the in-house gel electrophoresis system (Methods). ***C***, Plots of DNA bands from 12 wells (diluted to ~5ng/μl and measured following manufacturer's protocol) were analyzed with Agilent 2100 Bioanalyzer. ***D***, Plots of gDNA bands from 12 wells was analyzed with Fragment Analyzer (2μl of undiluted gDNA measured following manufacturer's protocol). ***E***, Distribution of gDNA band means (bp) assessed with the three different analysis methods. ***F***, Table listing mean size of gDNA fragment size from each well analyzed with the three different systems. The average of the DNA fragment mean and SDEV are shown in the last two columns of the table. The results show that analysis using the traditional Agilent 2100 Bionalyzer yield higher fragment sizes than using either the in-house system or Fragment Analyzer. The reasons for the differences, which are small, are not clear. These results show that the in-house very low cost agarose gel electrophoresis system is well suited for analysis of sheared gDNA fragments given that the bands are visualized with ethidium bromide (or Sybr green) staining.

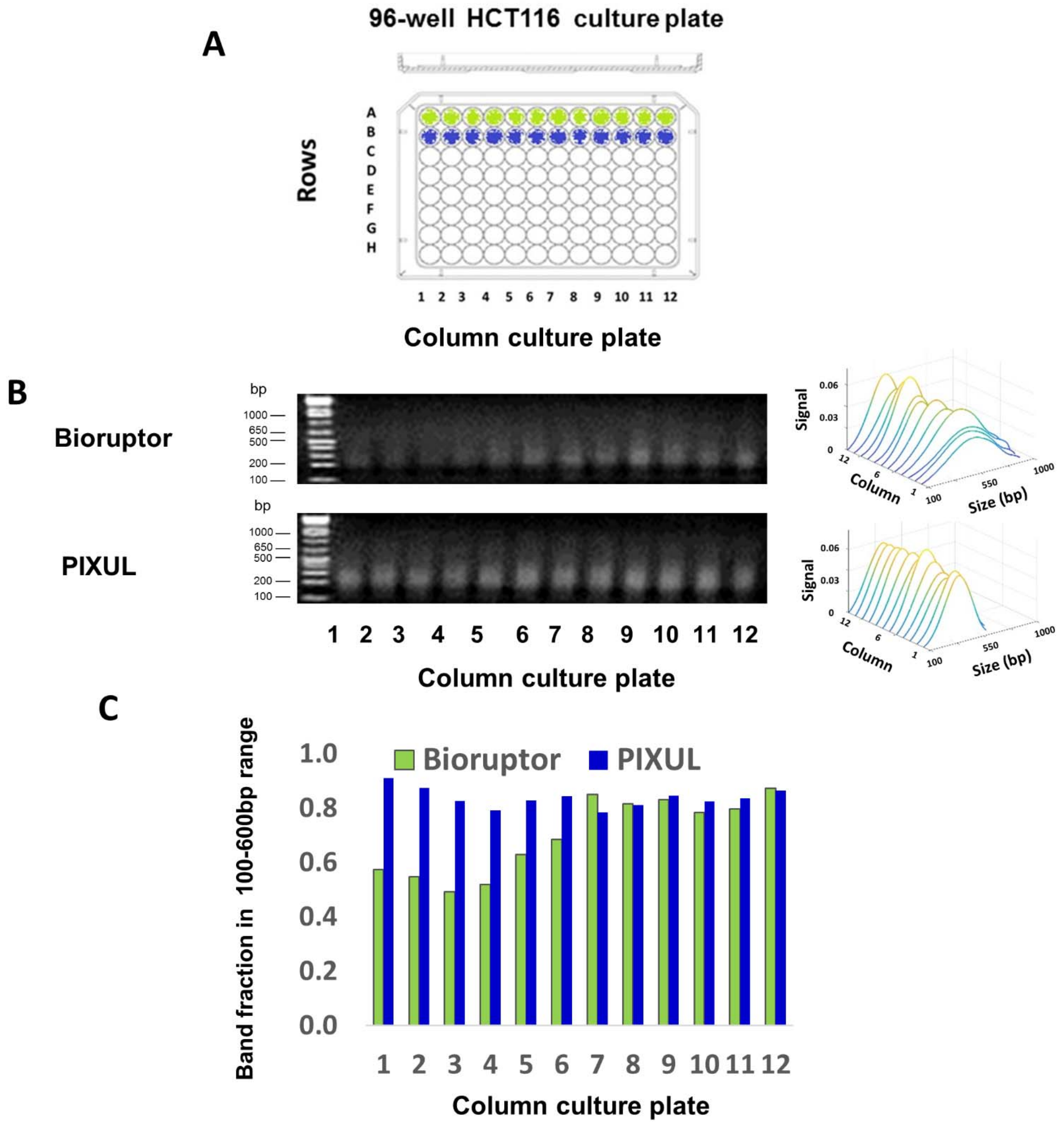
A
96-well HCT116 culture plate
Rows
A
B
C
D
E
F
G
H
1 2 3 4 5 6 7 8 9 10 11 12
Column culture plate
B
Bioruptor
PIXUL
bp
1000
650
500
200
100
1 2 3 4 5 6 7 8 9 10 11 12
Column culture plate
Signal
0.06
0.03
0
Column
Size (bp)
100
550
1000
C
Band fraction in 100-600bp range
Bioruptor
PIXUL
1.0
0.8
0.6
0.4
0.2
0.0
1 2 3 4 5 6 7 8 9 10 11 12
Column culture plate

Fig.S3. Comparison of shearing efficiencies of Bioruptor and PIXUL. Serum-deprived HCT116 96-well cultures. ***A***, Cells were crosslinked directly in the 96-well plate, quenched with glycine, and washed with PBS. PBS was then replaced with lysis buffer. Buffer is each well of row A was pipetted up and down several times, was then transferred to 0.5ml Eppendorf tubes and sheared using the Bioruptor. The rest of the plate was treated with PIXUL. ***B***, After proteinase K digestion and reversal of cross-linking, DNA was analyzed by agarose gel electrophoresis. Numbers to the left of the gels show sizes of selected ladder bands in base pairs (bp). Sheared fragments were analyzed by agarose gel electrophoresis image software (Methods) and shown here as waterfall plots (MATLAB) that contains best-fit curves in sequential order of culture plate column of samples 1 to 12. X- axis; band size in base pairs (bp). Y-axis; sample from a well of a given column. Z-axis; relative signal intensity of bands for given plate column. ***C***, The average size of Bioruptor sheared fragments was 394±64bp (69.9±14.1% in 200-600bp range) compared to 280±13bp (83.6±3.5% in 200-600bp range) with PIXUL (mean±SDEV, n=12).

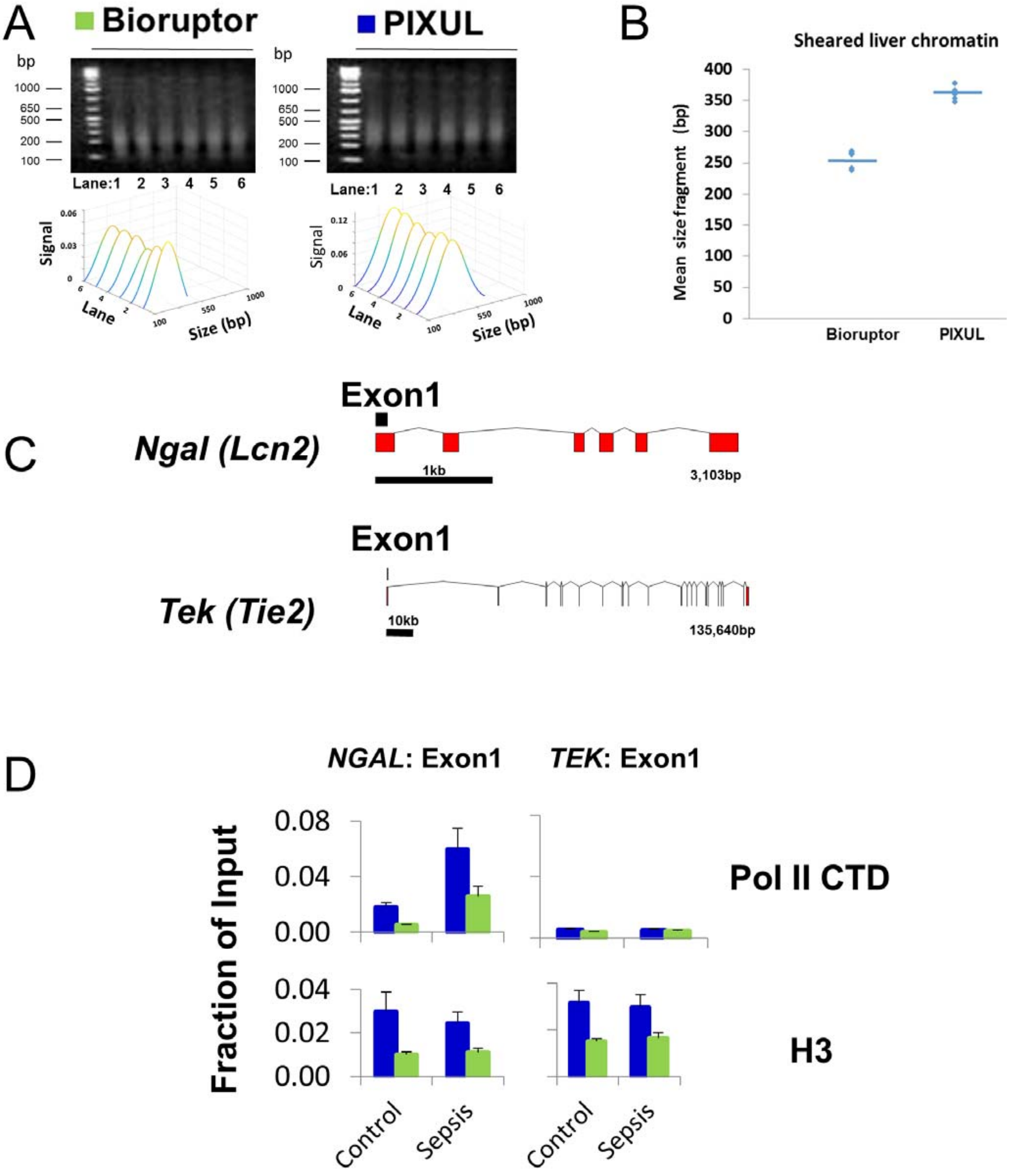
A
Bioruptor
PIXUL
bp
1000
650
500
200
100
Lane:1 2 3 4 5 6
Signal
Lane
Size (bp)
B
Sheared liver chromatin
Mean size fragment (bp)
Bioruptor
PIXUL
C
Exon1
Ngal (Lcn2)
1kb
3,103bp
Exon1
Tek (Tie2)
10kb
135,640bp
D
NGAL: Exon1
TEK: Exon1
Fraction of Input
Pol II CTD
H3
Control
Sepsis

Fig. S4. Matrix ChIP analysis of mouse liver chromatin prepared using PIXUL and Bioruptor. Fragments of flash frozen mouse livers were cross-linked and then sheared either in a 96-well plate using PIXUL (12min) or Bioruptor (45min). ***A,*** Agarose gel electrophoresis of sheared chromatin after proteinase K digestion and reversal of cross-linking. Numbers to the left of the gels show sizes of selected ladder bands in base pairs (bp). Sheared fragments were analyzed by agarose gel electrophoresis image software (Methods) and shown here as waterfall plots (MATLAB) that contains best-fit curves of samples 1 to 12 in sequential order, X- axis; band size in base pair (bp). Y-axis; sample from a well of a given column. Z-axis; relative signal intensity of bands for given lane on the gel. ***B***, Average size of sheared fragments. ***C,*** cartoon showing location of PCR primers along the genes. ***D,*** Sheared chromatin samples from control and septic mice livers (ref 40) were analyzed by Matrix ChIP with Pol II and H3 antibodies. Data represent mean$\pm$SEM (n=6 mice) expressed as a fraction of input. The results show consistent shearing of liver chromatin with either Bioruptor or PIXUL. Bioruptor yielded smaller fragments (by ~100bp). Matrix ChIP signal was higher with PIXUL (blue) compared to Bioruptor (green).

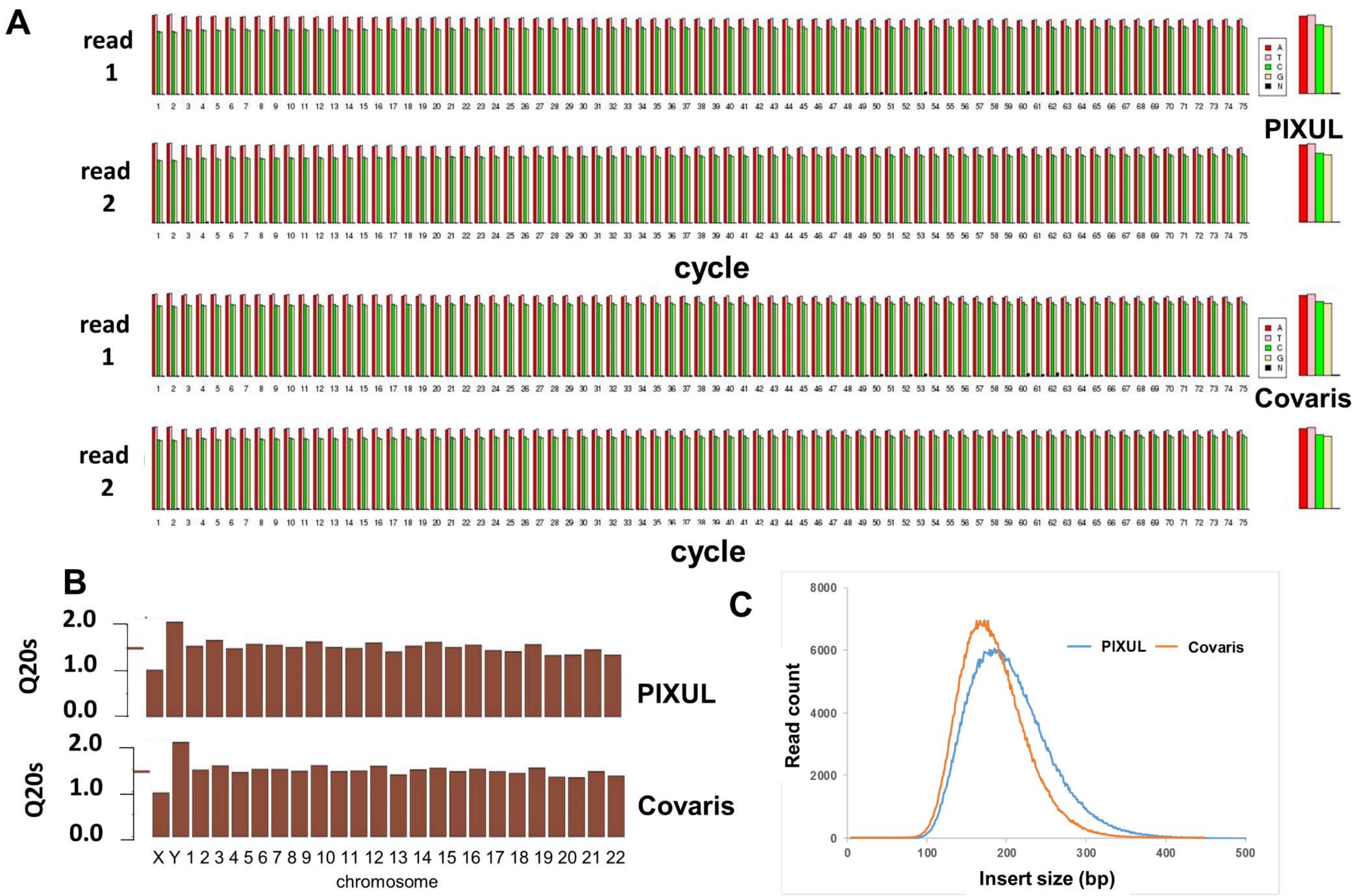

D

	PreCapture Yield	PreCapture Fragment Size	Insert Size- Sequencing	On Target
PIXUL	30ng/ul	340.5bp (+/- 10.3)	203.5bp (+/-3.4)	68%
Covaris	29.2ng/ul	316bp (+/- 1.8)	183.4bp (+/1.6)	72%

Fig. S5 Comparison of exome sequencing libraries prepared from human genomic DNA sheared by either PIXUL or Covaris LE220 Ultrafocused Sonicator.

Procedure: Four biological replicates consisting of 500ng gDNA (Promega, Madison, WI) were sheared in an off-the shelf 96-well plate using PIXUL (18min) or in a 96 well AFA microtube plate (Cat. 520168) using the Covaris LE 220 Ultrafocused Sonicator (450 watts, Duty Cycle 20%, 1000 cycles per burst for 3.5 min with dithering), (Covaris Inc, Woburn, MA). A 2X AMPure XP bead (Cat. A63882, Beckman Coulter, Indianapolis, IN) cleanup followed shearing to remove the smallest fragments, as well as to normalize sample volumes. Shotgun library was prepared using the Kapa HTP Library Prep Kit (Cat. KK8235, Roche, Indianapolis, IN) according to the manufacturer's instructions. End Repair was done by dA-taiing, adapter ligation, a dual SPRI size selection cleanup and 6 cycles of PCR using the Kapa HiFi Polymerase (Cat. KK2616). Adapters contained dual 8bp indices to facilitate multiplexing. The Quant-it dsDNA Assay Kit (Cat. Q33120I, Invitrogen, Carlsbad, CA) and the Agilent 2100 Bioanalyzer (Santa Clara, CA), were used to determine library quantity and quality, respectively. Libraries were pooled equally by mass into a single 8-plex capture pool, which hybridized for 64 hours with the Nimblegen V2 Exome probe set (Cat. 05860504001, Roche, Indianapolis, IN). Exome capture was followed by a stringency wash using the Nimblegen Hybridization and Wash kit (Cat. 05634253001) according to the manufacturer's recommendations. The final library pool was amplified a second time using the Kapa HiFi Polymerase in order to generate sufficient mass for sequencing. The final library pool was loaded on an Illumina MiSeq, using V3 chemistry and paired end 75bp reads, targeting 10 million reads for the pool (Illumina, San Diego, CA).

Results: Comparison between PIXUL and Covaris instruments are shown in the following panels: ***A***, Base distribution (A, *red*; T, *pink*; C, *green*; G, *yellow*) by sequencing cycle. The colored bars on the right expanded view to show order of the bases. ***B***, Mean quality of the base calls across the human chromosomes. ***C***, Distribution of library insert sizes. ***D***, Library construction and Sequencing metrics. PreCapture Fragment Size was determined using the Agilent 2100 Bioanalyzer. Sequencing Insert Size was determined by mapping to Hg19. The percent of reads on target is defined as the number of reads overlapping the Nimblegen V2 Exome probe region. This comparison shows that the yield and insert size are comparable for exome sequencing libraries prepared from gDNA sheared by either PIXUL or Covaris instruments. Panels *A* and *B* demonstrate that shearing by PIXUL produces fragments that are not biased by base composition. Shearing by PIXUL resulted in library inserts that were approximately 20bp longer than library inserts resulting from Covaris shearing. This likely led to a decreased number of reads mapping to the V2 exome target, as the sequencing read length was not sufficient to cover the entire insert.

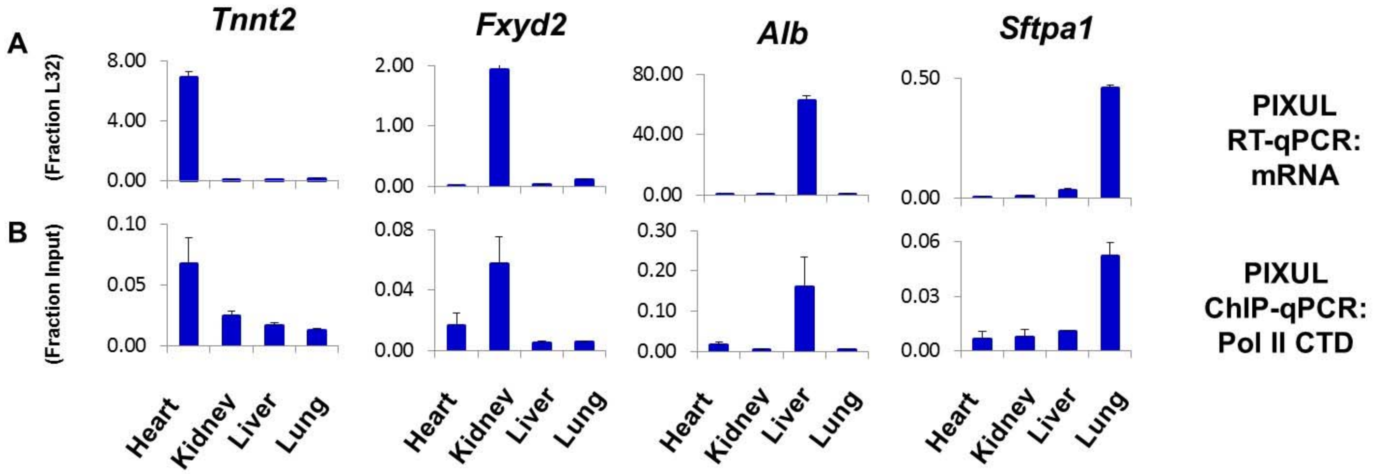

Fig.S6. Parallel PIXUL-RNA and PIXUL-ChIP analysis of mouse heart, kidney, liver, and lung. ***A***, Pieces of frozen heart, kidney, liver and lung samples well placed in wells of columns in 96-well plate containing 100µl TRIZOL and were treated in PIXUL (1min). RNA was isolated, reversed transcribed (using oligo dT) and analyzed by qPCR. Data represent mean+SEM (n= 3 mice) expressed as a ratio to the transcript levels of housekeeping ribosomal protein gene, L32. ***B*****,** Pieces from the same frozen organs as in ***A*** were cross-linked in wells of a 96-well plate and then sonicated in PIXUL (16min). PIXUL-sheared chromatin samples were simultaneously analyzed for Pol II levels at indicated organ-specific genes using Matrix ChIP. ChIP DNA was analyzed by qPCR. Data represent mean+SEM (n=3 mice) expressed as a fraction of input. These results demonstrate that PIXUL can be used to simultaneously processes samples from several organs for both chromatin and RNA isolation.

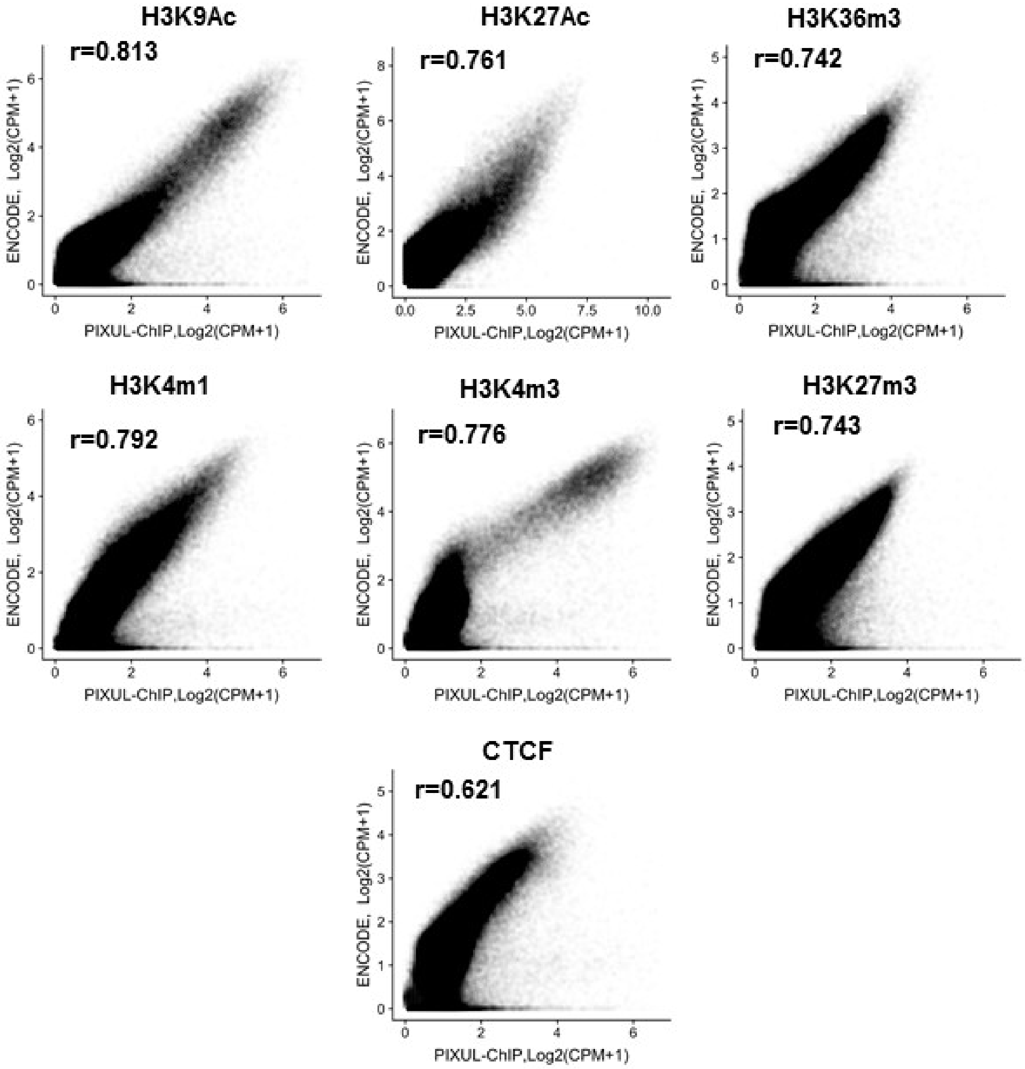

Fig. S7. Scatter plots of comparative analysis of PIXUL-ChIP-seq and ENCODE data sets. HCT116 cells were grown to the density of ~200,000 cells per well, cross-linked, and sonicated using 96-well PIXUL. ChIP was performed with the indicated antibodies and libraries were generated from a single PIXUL well using Active Motif's Low Cell ChIP-Seq Kit. Libraries were sequenced on a NextSeq 500 (Methods). deepTools (ref 46) was used for each set of ChIP-seq data to count the number of reads for each 5kb-sized bin across the human genome for ENCODE data and PIXUL-ChIP-seq data. Read counts were normalized in each bin to total read counts in each file. empty bins were filtered out, and plotted the normalized read counts (log2 counts per million mapped reads) comparing ENCODE and PIXUL-ChIP-seq data. Each dot represents a genomic bin. r = Pearson's correlation coefficient. Scatter plots demonstrate good correlation between PIXUL-ChIP-seq and ENCODE datasets. The differences between PIXUL-ChIP-seq and ENCODE data sets may reflect use of ChIP antibodies from different sources, growth conditions and the lower number of HCT116 cells (~200,000 for PIXUL-ChIP-seq) compared to ENCODE (>10^6).

TABLES

Table. S1. Antibodies

**Antibody**	**Assay**	**Catalog No.**	**Source**	**Manufacturer**
Pol II CTD (4H8)	ChIP-qPCR	Sc-47701	Mouse monoclonal	Santa Cruz
pRafB	ChIP-qPCR	sc-28006r	Rabbit polyclonal	Santa Cruz
pERK1/2	ChIP-qPCR	MA5-15173	Mouse monoclonal	ThermoFisher
H3K27Ac	ChIP-qPCR	39133	Rabbit polyclonal	Active Motif
H3K27m3	ChIP-qPCR	61017	Mouse monoclonal	Active Motif
H3K4m1	ChIP-qPCR	OAAH00066	Rabbit polyclonal	Aviva
H3	ChIP-qPCR	PA-16183	Rabbit polyclonal	ThermoFisher
H3K4m1	ChIP-seq	39297	Rabbit polyclonal	Active Motif
H3K4m3	ChIP-seq	39159	Rabbit polyclonal	Active Motif
H3K9ac	ChIP-seq	91103	Recombinant	Active Motif
H3K9m3	ChIP-seq	39161	Rabbit polyclonal	Active Motif
H3K27ac	ChIP-seq	39133	Rabbit polyclonal	Active Motif
H3K27m3	ChIP-seq	39155	Rabbit polyclonal	Active Motif
H3K36m3	ChIP-seq	61101	Rabbit polyclonal	Active Motif
CTCF	ChIP-seq	61311	Rabbit polyclonal	Active Motif

Table. S2. qPCR primers

Species	Gene	Purpose	Forward	Reverse
Human	*Egr1* -15kb	ChIP	GAGGCACTCTGCTCACCAAA	GATGCCTGCGAGGATGGAAA
Human	*Egr1* Exon1	ChIP	AGCTCTCCAGCCTGCTCGT	GGTAGTTGTCCATGGTGGGC
Human	*L32* Exon2-Exon3	RT	AGTTCCTGGTCCACAACGTC	TTGGGGTTGGTGACTCTGAT
Human	*NR4A3* Exon1	ChIP	CACTTTGCAACGCTGACG	AGAGCACAGCCGAGAGGTT
Human	*OCT4* Distal enhancer	ChIP	GAGGATGGCAAGCTGAGAAA	CTCAATCCCCAGGACAGAAC
Human	*OCT4* Exon5	RT	AGGGAAGGTGAAGTTCAATG	AGTGTGTCTATCTACTGTGTCC
Human	*OCT4* Proximal enhancer	ChIP	TCTGTTTCAGCAAAGGTTGGG	TTGGTCCCTACTTCCCCTTCA
Human	*OCT4* Proximal promoter	ChIP	GCAAACATCCTTCGCCTCAG	GTGAAATGAGGGCTTGCGAAG
Human	*TBX* Exon8	ChIP/RT	GTTAGCCCTTCCTTTTGCAG	GGCTGTCTCTAGCACATTCT
Human	*UBE2b* TSS	ChIP	CTCAGGGGTGGATTGTTGAC	TGTGGATTCAAAGACCACGA
Mouse	*Alb* Exon 15	RT	TGAAGACTCAGGACTCATCTTTT	CAGCACAGAGACAAGAAGTC
Mouse	*Alb* Exon1	ChIP	GTGGGTAACCTTTCTCCTCC	CTTCTCGGCGAAACACAC
Mouse	*Fxyd2* Exon 6	RT	CAGCTTCTCTAACACCCCAC	GATTCATTGAAAACCAGGGGG
Mouse	*Fxyd2* Intron1	ChIP	CCAGTGGCTCTCTTAGAAAAT	TCCAACTTCTGCTATTCTGCT
Mouse	*L32* Exon2	RT	TTAAGCGAAACTGGCGGAAAC	TTGTTGCTCCCATAACCGATG
Mouse	*Ngal (Lcn2)* Exon1	ChIP	AGTTCTGAGTTGAGTCCTGG	CCTAGTAGCTGTGGAAACCA
Mouse	*Sftpa1* Exon 6	RT	GACTGATCACATGCTGCCTA	GACTGATCACATGCTGCCTA
Mouse	*Sftpa1* Exon1	ChIP	GGTATCCACCAGTGTATGGG	GTTAGTCGTACGCAGTACCT
Mouse	*Tek (Tie2)* Exon1	ChIP	GTTGTTGAAAGCTTCCCAGG	ACTAAGCCGGCTAAAGAGTC
Mouse	*Tnnt2* Exon1	ChIP	ATGAGGGATTTGTTCCTGACA	CTTGAAAGGGCCATGGATTT
Mouse	*Tnnt2* Exon15	RT	CCAATGCAGACTCCTGTTTG	TGGCTTTTTATTGCTGGCAT

Table S3. PCR SYBR Green standard curves values

$Conc = 2^{m+CT+b}$						DNA standards concentrations			
						**10ng/μl**	**1ng/μl**	**0.1ng/μl**	**0.01ng/μl**
**Species**	**Gene**	**Site**	***m***	***b***	**R^2**	***CT***	***CT***	***CT***	***CT***
Human	*EGR1*	-15KB	-0.96142	22.83682159	0.99787192	20.46227	23.64252	26.97392	30.84474
Human	*EGR1*	Exon1	-1.0595	25.40899466	0.999866197	20.83387	23.96844	27.18473	30.21159
Human	*NR4A3*	Exon1	-1.01413	24.49731224	0.999362502	20.81794	24.18686	27.56750	30.60290
Human	*L32*	Exon2-3	-0.93066	19.64266572	0.995315966	17.41271	21.38755	24.51812	28.23455
Human	*OCT4(Pou5f1)*	proximal promoter	-1.12928	26.34763982	0.99674837	20.21958	23.51546	26.46326	29.01056
Human	*OCT4(Pou5f1)*	proximal enhancer	-1.25625	29.02511752	0.97647756	20.17253	23.41318	26.30604	27.81532
Human	*OCT4(Pou5f1)*	distal enhancer	-1.13125	25.89429515	0.998239316	19.88468	22.89773	26.04305	28.60736
Human	*OCT4(Pou5f1)*	Exon5	-1.03925	24.34699719	0.999062329	20.20588	23.38202	26.80543	29.70966
Human	*TBX3*	Exon8	-1.04737	24.22239438	0.999842049	19.96212	23.15491	26.22403	29.50969
Human	*UBE2b*	promoter	-1.06378	24.77485856	0.996099787	20.05486	23.31994	26.74722	29.28100
Mouse	*Alb*	Exon1	-1.31221	32.03308667	0.958453811	21.50841	24.93118	27.54442	28.72524
Mouse	*Alb*	Exon15	-1.55635	36.38543901	0.907640258	20.81847	24.26888	25.99595	26.70045
Mouse	*Fxyd2*	Intron1	-1.06565	26.16222801	0.999195162	21.36063	24.59639	27.80640	30.67320
Mouse	*Fxyd2*	Exon6	-1.02277	25.44273015	0.998903257	21.56976	25.00036	28.05856	30.85863
Mouse	*L32*	Exon2	-1.01492	23.40260438	0.998558065	19.74526	23.01627	26.56060	29.45840
Mouse	*Ngal*	Exon1	-1.31509	33.25715062	0.966129078	22.45833	25.64422	28.44519	29.65949
Mouse	*Sfpta1*	Exon1-Intron1	-1.10174	28.63563708	0.996611396	22.79856	26.18344	29.20587	31.80757
Mouse	*Sfpta1*	Exon6	-1.09733	26.99753569	0.991875297	21.40752	24.69304	28.07710	30.28844
Mouse	*Tek(Tie2)*	Exon1	-1.09742	26.68940967	0.99462805	21.14406	24.40414	27.70723	30.07891
Mouse	*Tnnt2*	Exon1-Intron1	-1.08061	25.71352579	0.997035426	20.59524	23.87461	27.13455	29.72528
Mouse	*Tnnt2*	Exon15	-1.1071	26.24016473	0.986737288	20.51744	23.79065	27.27450	29.22540

Table S4. Labware and kits

Supplier	Location	Catalog No.	Item Description	COST	COST/WELL-REACTION	
Corning	Oneonta, NY	3799	Costar 96-well clear round-bottom (used in PIXUL)	$2.36/plate	$0.02	/well
Applied Biosystems	Carlsbad, CA	4311971	MicroAmp Optical Adhesive Film (used in PIXUL)	$1.77/plate	$0.02	/well
Covaris	Woburn, MA	520052	microTUBE AFA Fiber Crimp-Cap 6x16mm (used in Covaris)	$127.50/25 tubes	$5.10	/ tube
Covaris	Woburn, MA	520168	96 microTUBE-50 AFA Fiber Plate (used in Covaris)	$459/plate	$4.78	/well
Agilent	Santa Clara, CA	5067-4626	High Sensitivity DNA Kit	$613/110 samples	$5.57	/sample
Agilent	Santa Clara, CA	DNF-492	Standard Sensitivity Large Fragment Analysis Kit	$1743/500 samples	$3.49	/sample
VWR	Radnor, PA	490003-794	Matrix ChIP 96-well semi-skirted conical polypropylene plates	$2.35/plate	$0.02	/well
Active Motif	Carlsbad, CA	53084	Low Cell ChIP-Seq Kit	$1235/16 rxns (kit)	$77.19	/rxn
Beckman Coulter	Indianapolis, IN	A63882	Agencourt AMPure XP Bead Kit	$5900/450mL	$450	/mL
Roche	Indianapolis, IN	KK8232	Kapa LTP Library Prep Kit	$1176/48 rxns (kit)	$24.50	/rxn
Invitrogen	Carlsbad, CA	Q33120	Quant-it dsDNA High-Sensitivity Assay Kit	$413.09/1000 samples (kit)	$0.41	/sample
Roche	Indianapolis, IN	05860504001	SeqCap EZ Exome V.3	$4200/4 rxns	$1,050	/rxn
